# Global and non-Global slow oscillations differentiate in their depth profiles

**DOI:** 10.3389/fnetp.2022.947618

**Published:** 2022-10-24

**Authors:** Sang-Cheol Seok, Elizabeth McDevitt, Sara C. Mednick, Paola Malerba

**Affiliations:** ^1^ Battelle Center for Mathematical Medicine, Nationwide Children’s Hospital, Columbus, OH, United States; ^2^ Princeton University, Princeton, NJ, United States; ^3^ Department of Cognitive Sciences, University of California, Irvine, Irvine, CA, United States; ^4^ Center for Biobehavioral Health, Abigail Wexner Research Institute, Nationwide Children’s Hospital, Columbus, OH, United States; ^5^ School of Medicine, The Ohio State University, Columbus, OH, United States

**Keywords:** slow oscillations, sleep, space-time, sleep EEG, machine learning

## Abstract

Sleep slow oscillations (SOs, 0.5–1.5 Hz) are thought to organize activity across cortical and subcortical structures, leading to selective synaptic changes that mediate consolidation of recent memories. Currently, the specific mechanism that allows for this selectively coherent activation across brain regions is not understood. Our previous research has shown that SOs can be classified on the scalp as Global, Local or Frontal, where Global SOs are found in most electrodes within a short time delay and gate long-range information flow during NREM sleep. The functional significance of space-time profiles of SOs hinges on testing if these differential SOs scalp profiles are mirrored by differential depth structure of SOs in the brain. In this study, we built an analytical framework to allow for the characterization of SO depth profiles in space-time across cortical and sub-cortical regions. To test if the two SO types could be differentiated in their cortical-subcortical activity, we trained 30 machine learning classification algorithms to distinguish Global and non-Global SOs within each individual, and repeated this analysis for light (Stage 2, S2) and deep (slow wave sleep, SWS) NREM stages separately. Multiple algorithms reached high performance across all participants, in particular algorithms based on k-nearest neighbors classification principles. Univariate feature ranking and selection showed that the most differentiating features for Global vs. non-Global SOs appeared around the trough of the SO, and in regions including cortex, thalamus, caudate nucleus, and brainstem. Results also indicated that differentiation during S2 required an extended network of current from cortical-subcortical regions, including all regions found in SWS and other basal ganglia regions, and amygdala and hippocampus, suggesting a potential functional differentiation in the role of Global SOs in S2 vs. SWS. We interpret our results as supporting the potential functional difference of Global and non-Global SOs in sleep dynamics.

## Introduction

Slow oscillations (SO, 0.5–1.5 Hz) are characteristic graphoelements of non-random eye movement sleep (NREM) associated with health, restorative properties of sleep and brain homeostasis (Tononi and Cirelli, 2006; Lee et al., 2015; Fultz et al., 2019). Occasionally, faster oscillatory rhythms (thalamo-cortical spindles and hippocampal ripples) emerge locked at specific SO phases. Properties of SOs like density and amplitude, and their coordination with faster sleep rhythms, have been connected to the improvement in memory performance that can occur across an epoch of sleep (Maquet, 2001; Marshall et al., 2006; Walker and Stickgold, 2006; Diekelmann and Born, 2010; Molle and Born, 2011; Ngo et al., 2013; Rasch and Born, 2013; Dudai et al., 2015; Ladenbauer et al., 2016; Ong et al., 2016; Papalambros et al., 2017; Mikutta et al., 2019).

When detecting these events in a sleep EEG, each SO can be found to occur in many (almost all) electrode locations at very short delays, or at only some locations. Thus, there is a spatial component to the presentation of SOs on the electrode manifold. SOs on an EEG are also known to present interesting time relations with other sleep brain rhythms. The nesting of faster rhythms within SOs is a measure based on reciprocal timing of the phase of the SO and the amplitude of fast rhythms, thus, it underscores the importance of understanding the timing of SOs paired to their spatial organization. Our research focuses on describing the organization of SOs as seen on the EEG electrode manifold, and studying the potential functional implications of patterns of differentiation of SOs in distinct space-time profiles.

In a recent analysis, we have shown that SOs establish windows of opportunity at phases preceding and following the SO trough during which information flow between distal electrodes (placed far away from each other) peaks (Niknazar et al., 2022). These phases of enhanced information flow captured times in the SO shape that indicated transition into a Down State (where the underlying network of pyramidal cells shows widespread hyperpolarization) and emergence to an Up State (organized sparse firing in the pyramidal cells initiated by a cascade of events in the network (Timofeev et al., 2000; Bazhenov et al., 2002)). The property of SOs to mediate specific times in which information flow among distant regions is enhanced suggest that SOs are particularly relevant to information processing during sleep, as NREM sleep has been identified as a state of overall loss of functional connectivity compared to wake or REM sleep. This again supports a relevance for the timing and spatial profiles of SO events as they create windows of opportunity for information flow.

In recent work (Malerba et al., 2019), we have introduced a data-driven methodology that captures the organization of SOs on the electrode manifold in space-time patterns. Our results showed that SOs can be classified on the scalp as Global, Local or Frontal, based on co-detection across multiple electrodes at short delay. Specifically, when SOs emerge as Global, they are detected in almost all electrodes at a short delay; Frontal SOs are detected at the frontal electrodes only in a small delay; while Local SOs are detected at only few electrodes, and with no particular specificity of location. Further analysis showed that Global SOs had larger amplitudes than other SO types, and showed enhanced coordination with sleep spindles. In a follow-up study (Niknazar et al., 2022), we reported that Global SOs support information flow at long ranges, with effective connectivity estimates that were strongly related to improvement in memory consolidation across sleep. Non-Global SO types did not show these properties nor relations with memory. Taken together, our research suggests that there is a potential functional relevance to the differentiation of Global SOs and non-Global SO types.

Since research on the functional role of SOs emphasizes their role in coordinating timing of activity across cortical and subcortical regions, in this work we were interested in studying the potential differentiation of SO types in cortical-subcortical regions, beyond their emergence on the scalp. In the current study, we compared the depth structure of Global and non-Global SOs (i.e., Local or Frontal) on the scalp in a dataset of natural full-night sleep. We chose to leverage this dataset of sleep EEG recordings of healthy young adults acquired with a 64-channels cap (details in methods) to detect SO events and analyze their space-time profiles, both on the scalp and in their cortical-subcortical estimates. In this analysis, we focused on assessing whether the cortical-subcortical presentation of Global and non-Global SOs differentiated within and across individuals, and identified which features of the depth profiles were most relevant to differentiating Global and non-Global SO activity before, after or around the trough.

First, we detected SOs and classified them according to their space-time profile presentation on the electrode manifold, applying the approach from (Malerba et al., 2019). The result replicated our previous findings that SOs present as Global, Local or Frontal in the scalp, and that the relative proportion of SO of Global SOs is different in light (stage 2, S2) vs. deep (slow wave sleep, SWS) NREM sleep. Then, we used source localization to construct a dataset of current source density (CSD) profiles for each SO, estimated with available software resources [Brainstorm (Tadel et al., 2011) combined with sLORETA (Pascual-Marqui, 2002)]. We embedded each depth profile in a matrix representation that averages the current found in each brain region and three main time intervals (before, during and after an SO trough). Using each SO as a point in a finite dimensional space, we labeled each point as Global or non-Global, according to their profiles on the scalp. To investigate the differentiation of Global/non-Global SO depth profiles within and across individuals, we analyzed this dataset with multiple machine learning (ML) algorithms, and identified the best performing classifiers. We used feature selection approaches to determine the elements of the SO depth profiles expected to be most relevant to Global/non-Global differentiation, and assessed the performance of generalized classifiers that were trained only on the most selective features in comparison to those trained on all features.

Our results demonstrated that multiple algorithms distinguish Global and non-Global SOs with high accuracy, both within and across individuals. This suggests that Global and non-Global SOs are structurally different in their current source density depth profiles. The most accurate classification was achieved by multiple algorithms based on k-nearest-neighbors principles, suggesting that univariate feature selection could appropriately identify the elements of the depth profiles most relevant to classification. During both S2 and SWS, the highest-ranking features according to univariate selection were in the time epoch around the trough, rather than preceding or following, and involved currents in cortex, thalamus, brainstem as well as the caudate nucleus within the basal ganglia. Currents in the amygdala, hippocampus, pallidum and striatal regions were relevant to classification for SOs in S2, but not in SWS. In addition, reducing the space to only the highest-ranked features did not affect performance in the high performing classifiers, confirming their relevance in differentiating Global and non-Global SO dynamics. Overall, our analysis further supports the structural and functional difference between Global and non-Global SOs, which is likely reflective of their differential role in information processing during NREM sleep.

## Materials and methods

### The sleep dataset

This work builds on a sleep polysomnography dataset collected in the Sleep and Cognition Lab laboratory, directed by Dr. Mednick. The data includes the EEG sleep night of 22 volunteers (9 females) with no history of psychological and neurological problems. All participants signed informed consent. This study was approved by the University of California, Riverside Human Research Review Board. EEG signals were acquired using a 64-channel cap (EASYCAP GmbH) with Ag/AgCI electrodes placed according to the international 10–20 System at a sampling rate of 1,000 Hz. Fifty-eight EEG channels were used for EEG signal recording, and the others were used for reference, ground, and other biosignals, including electromyography, electrooculography, and electrocardiography. EEG signals were re-referenced to the contralateral mastoid (A1 and A2) and down-sampled to 500 Hz after the recording. Raw data were visually scored in 30-s-long epochs into Wake, Stage 1, Stage 2, SWS, and REM sleep according to the Rechtschaffen & Kales’ manual (Rechtschaffen, 1968) using HUME (Saletin, 2015), a custom MATLAB toolbox. The basic sleep characteristics found in the dataset are reported in Supplementary Table S1.

### Slow oscillations detection and space-time profiles

SO detection was performed on artifact-free epochs scored as S2 and SWS. SO detection and space-time profile identification was conducted in Matlab with custom scripts. To detect the presence and timing of each SO event in any given electrode, we first applied a detection algorithm that we used in our previous work (Malerba et al., 2019) and closely followed the criteria introduced by Massimini et al. (2004), and was initially introduced in Dang-Vu et al. (2008). The following description matches the one reported in methods in (Malerba et al., 2019). In short, the EEG signal was filtered in the 0.1–4 Hz range, and candidate portions of the signal between subsequent positive-to-negative and negative-to-positive were listed as possible SOs. These events were only considered SOs if the following criteria were satisfied: 1) the wave minimum was below or equal to 80 uV, 2) the range of values between maximum and minimum voltage was at least 80 uV, 3) the time between the first and second zero crossing in the data had to be between 300 ms and 1 s, and 4) the total duration of the candidate event was at most 10 s. The pool of candidate SO events satisfying the parameters were further screened to remove potential artifacts, by computing the amplitude at trough referenced to the average of the signal ±10 s around the minimum. Events at one electrode which showed an amplitude size of 4 SDs above the mean of all events detected at that electrode were discarded, and a secondary distribution of amplitudes including all events from all electrodes of a subject was created, and again events with amplitude above 4 SDs from the mean were discarded.

After SO detection, we performed clustering of SO co-detections to reveal the space-time patterns of SOs in this dataset. We used the same procedure as in (Malerba et al., 2019). In short, a co-detection matrix was built for S2 and one for SWS, where each SO at each electrode was used to generate a binary array the length of all available head electrodes, with values of zero/one depending on whether an SO was found at a ±400 ms time delay from the SO trough (1 corresponding to successful co-detection). The co-detection binary matrix was then analyzed with k-means clustering using Hamming distance, with 200 replicates and a maximum iteration of 10,000 with the option of dropping empty clusters and preserving the default setting of adaptive initialization with kmeans++ (leveraging the k-means function in Matlab, TheMathworks). Counts of detected SOs in S2 and SWS per each participant, also separating Global and non-Global SOs, are reported in Supplementary Table S2.

### Statistical comparisons of Global non-Global slow oscillations current source density in regions

For comparisons on data in Figure 3, we only compared within a given time bin and sleep stage at a time. We use a two-factor, within-subject repeated measures ANOVA, with factors region by SO type and dependent variable the CSD values (a.u.). In post-hoc analysis, we used Wilcoxon signed-rank test to compare CSD in Global vs. non-Global SOs at each region. Since we have 17 regions in each group, and we are not comparing crossing across groups, we have 17 possible pairs to compare, taking our Bonferroni corrected threshold to 0.05/17 = 2.9e-3. Pairs that are significantly different are marked with an asterisk.

### Slow oscillations current source density estimation

The channel data for the time of the SO were imported into Brainstorm (Tadel et al., 2011). We used a default anatomy distributed with Brainstorm, the MNI ICBM152 package, which has high compatibility with different features within Brainstorm. We built a mixed head model with both cortex and sub-cortical substructures on the MRI images. Regions included: neocortex (labeled Cortex in figures), hippocampus, nucleus accumbens, amygdala, the brainstem, caudate nucleus, putamen, pallidum, and thalamus. Source estimation was performed at each time point of interest by fitting current dipoles in a fixed three-dimensional grid composed of voxels with 15,002 vertices for neocortex and 5095 vertices for sub-cortical structures (consistent with Brainstorm settings). In each vertex, we consider a single dipole with its orientation perpendicular to the cortical vertex. This approach constructs a total of 20,097 dipoles on 20,097 voxels. We used the boundary element method (BEM) OpenMEEG (Kybic et al., 2005; Gramfort et al., 2010) to compute the lead field matrix (forward modeling). We then used the minimum norm method to estimate a solution to the linear inverse problem, applied with the identity as noise covariance matrix (i.e., we did not model the noise component). This minimized the assumptions we imposed on our estimates, since the current dataset was limited to scalp EEG data, and hence we only had information on the noise covariance elements already present in the EEG data. To obtain a final CSD value in each voxel, we applied Brainstorm with the option of using standardized low-resolution brain electromagnetic tomography (sLORETA (Pascual-Marqui, 2002). This step minimizes bias in the source estimates and allows for comparison of CSD profiles across individuals. One advantage of our applied inverse modeling is that the solutions produced by Brainstorm with sLORETA are expressed in a combination of the instantaneous EEG data with a linear kernel (Wendel et al., 2009), which is not dependent on the specific voltage values in the EEG data, but only on electrode placement. As a result, we can calculate the CSD estimate at a large number of time points just by extracting the linear kernel one single time, and then applying it to the EEG datapoints of interest.

### Implementation of machine learning models

To study the classification accuracy of multiple algorithms, we leveraged a supervised learning application in Matlab (TheMathworks, R2021a) called Classification Learner, which is included in the Statistics and Machine Learning toolbox. This app integrates all commonly used tools regarding classifications: exploring data, selecting features, training data, and assessing the classifiers. The version we used includes 30 classification models, all used in our analysis. The classifiers (algorithms) are organized in 8 categories: Decision Trees (DT), Discriminant Analysis (DA), Logistic Regression classifiers (LR), Naïve Bayes classifiers (NB), Support Vector machines (SV), Nearest Neighbor classifiers (KN), Ensemble classifiers (ES), and Neural Network (NN) classifiers. Of note, ES classifiers combine multiple classifications methods, which tends to improve accuracy at the expense of rapidity.

While training time for any classification learner depends mainly on the complexity and the size of the data and algorithm it implements, it also depends on hardware (CPU and physical memory) and software of the machine where it is run. Regardless of data size, some learners like DT and DA algorithms ran fast. In our study, all SV learners, some NB classifier (Kernel), Cubic KNN, and Subspace KNN learners took significant time. As the size of data increased, some classifiers showed linear increase in time, others an exponential one. Of note, beyond a given data size, there is a loss of capacity in the physical memory that leads to the OS swapping to the virtual memory. This swapping time can add to the training time, and can result in thrashing when the data size is too large. To avoid this circumstance, a user replicating our approach should consider emptying the physical memory as much as possible before training SWS data.

With our approach, we obtained 30 classifiers for each 22 individual sets of Global and non-Global SOs, resulting in a total of 660 classifiers in S2 (and the same amount in SWS). This was possible because enough Global and non-Global SOs were sourced in each participant (in S2 and SWS, evaluated separately, see Supplementary Table S2).

To estimate classifier performance, we chose non-exhaustive approaches due to dataset size. We used cross-validation with 5-folds (i.e., building 5 groupings of 20% of the data iteratively used for testing and averaging the resulting accuracy) when testing classifiers on datasets that were constrained to SOs acquired from one individual (Figures 4, 5). We used holdout validation with 25% of the data held out when training classifiers on the comprehensive datasets that combined SOs from all participants (Figure 7). We were interested in evaluating performance beyond the training domain. Hence, the output of testing each trained classifier on the full dataset was organized in “true positives” (TP, correct classification of Global SOs), “true negatives” (TN correct classification of non-Global SOs) and corresponding “false positives/negatives” (FP, FN) when classification was incorrect for Global and non-Global SOs, respectively. Due to the not-balanced nature of the labeling in our dataset (in each individual we found more non-Global than Global SOs, consistently with (Malerba et al., 2019)) we considered performance estimators that could account for the imbalance, and found that the Matthews Correlation Coefficient (MCC) is highly informative for binary classification (Chicco et al., 2021a; Chicco et al., 2021b). We then chose MCC as our metric for performance across this work, and calculated it from the binary confusion matrix according to the following formula:

MCC=(TP*TN−FP*FN)(TP+FP)*(TP+FN)*(TN+FP)*(TN+FN) ; with the exception that if the argument of the square root at the denominator is not strictly positive, MCC defaults to 0. This index ranges from −1 to 1, with positive values closer to 1 indicating highest performance.

## Results

### Global and non-Global slow oscillations dataset: Analytical framework and preliminary comparisons

**FIGURE 1:**
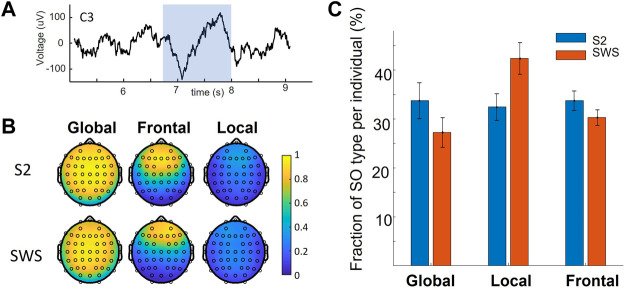
Global and non-Global Slow Oscillations on the scalp. **(A)** An example of a detected SO in a centrally located EEG channel (C3, on the left hemisphere). The shaded range marks the detected SO. **(B)** Average of all co-detections found for all SO events that are labeled within a given cluster, mapped on the scalp. Values of 1 indicate that co-detection at that electrode was found in all SO events grouped in that given cluster. Vice-versa, a near-zero value shows that only very few SOs in that cluster had a co-detection at the evaluated location. Note the replication of results found in our previous work, where SOs can be labeled as Global, Frontal or Local depending on their co-detection on the scalp. **(C)** The fraction of SO type across individuals in this dataset (mean and standard error of the mean) in the two sleep stages of interest. Note the decrease in fraction of Global SOs in SWS compared to S2 sleep, mirrored by an increase in Local SOs in SWS compared to S2. Statistical analysis showed a significant difference in the fraction of Local SOs in S2 vs. SWS.

This study leverages a dataset that contains full-night sleep recordings from 22 adult volunteers, with sleep EEG acquired with 64 leads. This dataset was acquired by the Mednick group for a different purpose and is being re-analyzed in our retrospective analysis. To identify SOs algorithmically at each channel in NREM stages S2 and SWS and classify each SO as Global, Local or Frontal, depending on their space-time profile on the scalp we used the same methodology as introduced in our previous work (Malerba et al., 2019). Full details of these procedures are reported in the methods. Briefly, we used established algorithms to detect each SO event, and for each event we identified which electrodes also showed a detection within a short delay (400 ms before or after the SO trough). The resulting co-detection binary matrix was then clustered with k-means clustering, with parameters for the algorithms identical to the ones we used in our previous work. The analysis showed a replication of our published result in this new dataset, where we found that data-driven analysis of scalp co-detection reveals three SO types: Global, Local and Frontal, separately in both sleep stages (Figure 1), and that the proportion of Local SOs are different in S2 and SWS (a two-factor ANOVA found an effect of sleep stage by SO type interaction, post-hoc analyses established a difference in the fraction of Local SOs in S2 vs. SWS, with same comparison for Global SOs being close but not reaching statistical significance after Bonferroni correction). Thus, the property of being Global or not Global (i.e., Local or Frontal) is a characteristic that emerges in a data-driven way in both sleep stages independently, but does not emerge as an identical proportion. This differentiation supports our rationale to analyze the two sleep stages separately at every step of this study.

Since our previous work (Niknazar et al., 2022) has shown a selective relevance for Global SOs in supporting long-range communication in specific windows of opportunity around the SO trough, we were interested in studying the characteristics of Global SOs that differentiate them from other SOs and in focusing on time epochs that were relevant for this Global SOs role in directional information flow (around the trough, and preceding or following the SO trough). To compare the space-time profiles of Global SOs and those of all other SOs, we grouped Frontal and Local SOs in one category, labeled non-Global SOs. Next, we needed to establish a framework to analyze how Global and non-Global SOs differentiate in cortical and sub-cortical regions in epochs around the SO trough that we showed were important for information flow. This required estimating a current source density (CSD) depth profile for each SO that included cortical and subcortical regions, and using those profiles at specific times of interests for comparative analysis.

**FIGURE 2:**
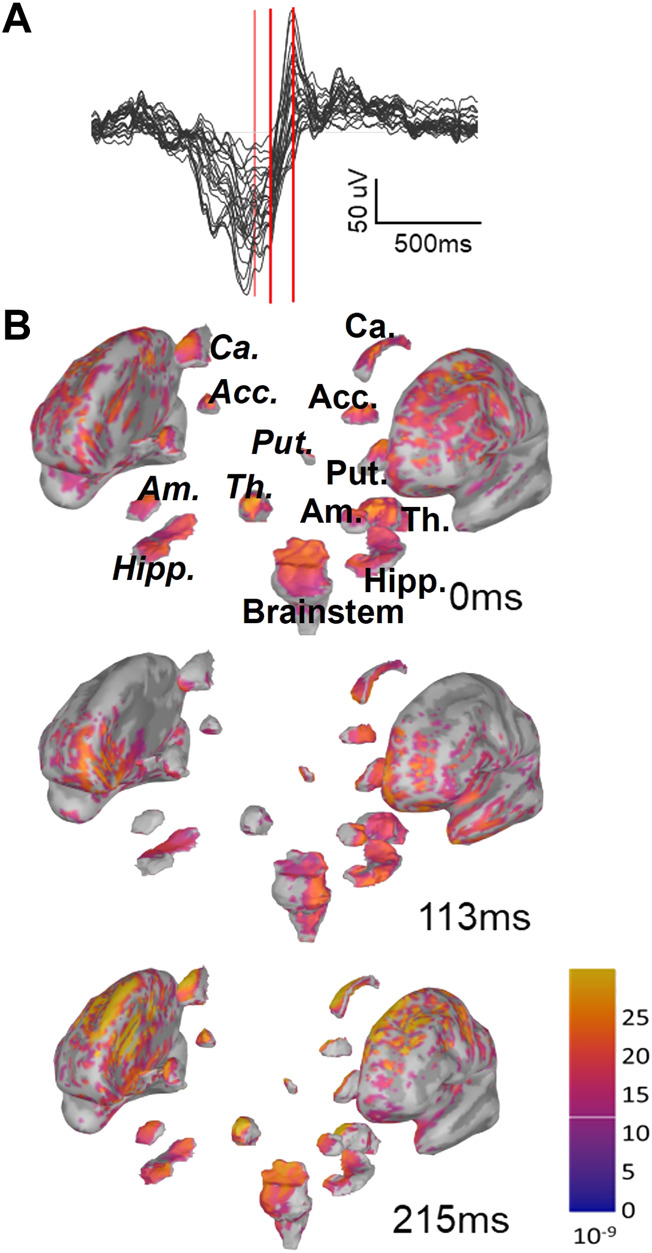
Instantaneous representation of current sources by Brainstorm. **(A)** One example of Global SOs EEG butterfly plot (all channels superimposed). This is the type of data that we insert in Brainstorm to estimate current sources for each SO. The three red bars mark three time points (trough, 113 ms after trough and 215 ms after trough). **(B)** Example of the graphic representations returned by Brainstorm with sLORETA for the same SO as in A at the three time points marked in A with red bars. Note that, as the up-state emerges, the overall current density increases and gets more widespread. For one of the plots we have labeled the sub-cortical regions visualized in this plot, using italics for regions in the right hemisphere. Abbreviated labels are as follows: Acc, nucleus accumbens; Ca, caudate neucleus; Put, putamen; Am, amygdala; Th, thalamus; Hipp, hippocampus.

To achieve CSD estimates for each time instant in a given SO, the EEG data were imported into Brainstorm (Tadel et al., 2011), a software designed to reconstruct current sources from EEG recordings (details in Methods). Our estimate included neocortex (labeled Cortex) and hippocampus, nucleus accumbens, amygdala, the brainstem, caudate nucleus, putamen, pallidum, and thalamus. We consider all regions as split into their left and right components, except the brainstem, resulting in 17 total regions. In Figure 2 we show one example of the depth profile of a Global SO at three time points starting from the trough and proceeding toward the peak of the up state. Note that as the SO transitions from Down to Up state, current appears to propagate to more regions and shows larger magnitudes. Applying this approach, we constructed an initial collection of voxel-by-time CSD values for each SO in our dataset, composed of 251,395 SOs in S2 (97,428 of them Global and 153,967 non-Global) and 921,076 SOs in SWS (322,135 of them Global and 598,941 non-Global), see Supplementary Table S2.

We then used this three-dimensional representation of SO dynamics in our analysis. Noting the high volume of datapoints (the SOs), and large complexity of the voxel-by-time representation of each SO, we chose to study the comparison between Global and non-Global SOs depth profiles algorithmically, with machine learning. To reduce complexity in the spatial component, we grouped voxels allocated to the same brain region and averaged the CSDs within them. The time component was organized in three time bins, each 200 ms long, with one time bin encompassing the (−100, +100) ms interval around the SO trough (“trough time”), one time bin at (−300,−100) ms from the trough (the “pre-trough”) and a corresponding time bin at (100,300) ms from the trough (the “post-trough”). Hence, CSD values were averaged across each time bin and across each sub-cortical structures to build matrices of 17 regions by 3 time bins, which can also be thought of as points in 51-dimensional space with trivial Euclidian distance (R^51^). Examples of the distribution of CSD values in each region and time epoch for one individual are shown in supporting Supplementary Figures S1,S2.

**FIGURE 3:**
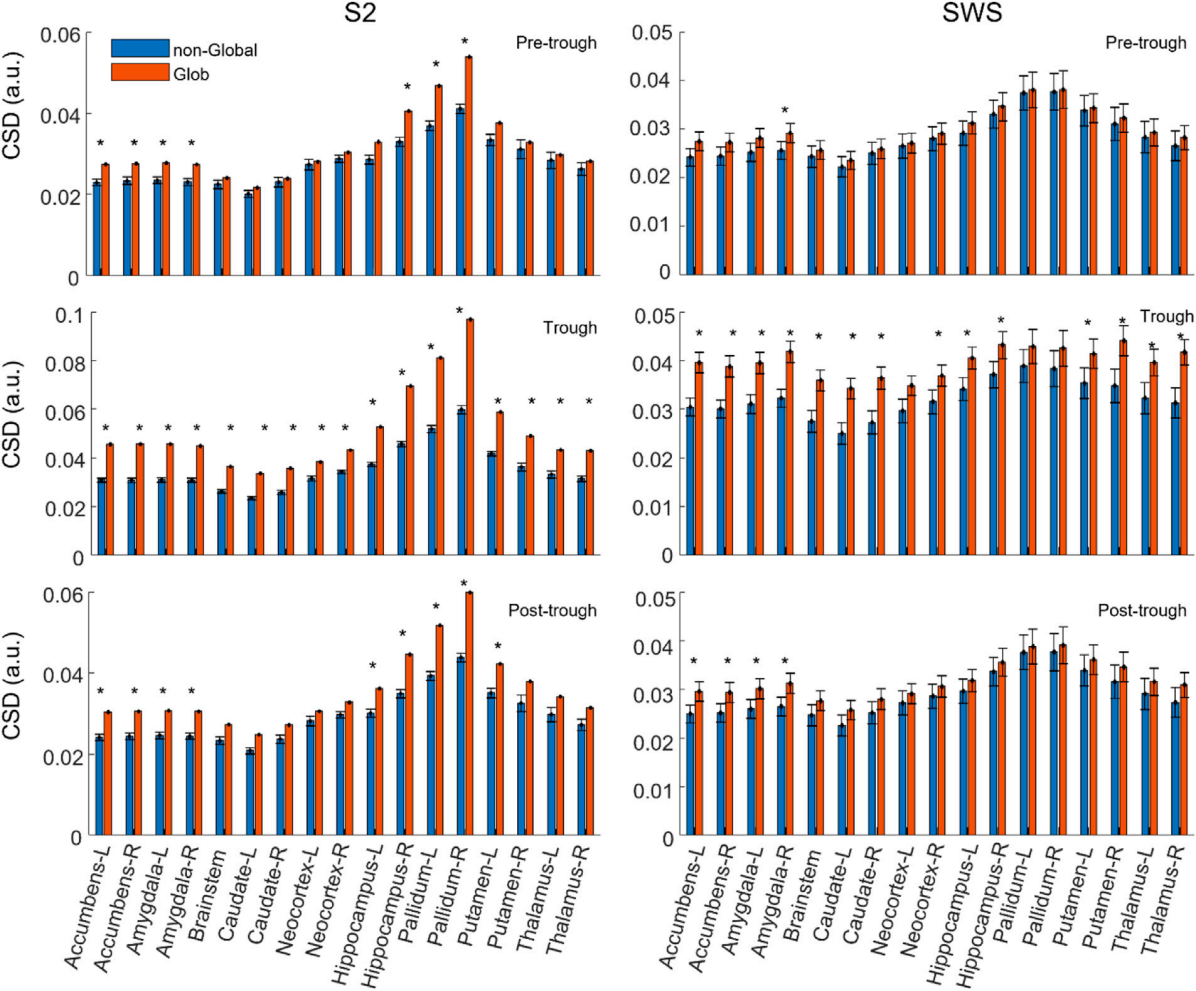
On average, the current density in each region is slightly larger in Global SOs compared to non-Global SOs. Average CSD value in each feature (region by time bin), comparing Global and non-Global SOs. CSD is reported as mean across all participants, and error bars mark the standard error of the mean. Labels for each region include its name and its hemispheric placement, L for left and R for right, except for the brainstem, which is not considered as separated between hemispheres. All considered regions are shown. Values for Global SOs are shown in red/orange, non-Global SOs are shown in blue (legend on top left side of figure). Plots on the left column refer to SOs found during S2, right column to SWS. Plots in the top row refer to CSD values in the Pre-trough time bin, middle row refers to the Trough time bin and bottom row to the Post-trough time bin (see labels on the top right corner of each plot).

To estimate initial systematic differences in the depth profiles of Global and non-Global SOs, we compared the average Global and non-Global SOs CSD value across participants in each region-time component. Figure 3 shows that average CSD values are relatively higher in Global SOs compared to non-Global SOs across individuals at all regions and times considered, both in S2 and SWS. Statistical comparisons showed that at times around the trough Global SOs had larger average CSD than non-Global SOs in all or almost all regions, both in S2 and SWS. In time epochs before and after the trough, the difference between average values was less pronounced in most regions, with a slight prevalence of subcortical differentiation at non-trough epochs in S2 for hippocampus, pallidum, nucleus accumbens, and the amygdala (see Supporting information for exact *p*-values). Results in Figure 3 suggest that the deep profiles of SOs may differentiate the scalp classification of Global vs. non-Global, confirming our intuition that this dataset should support structural differentiation of Global and non-Global SOs. It also showed that SOs in SWS and S2 were not differentiating in their Global and non-Global profiles in a uniform manner, highlighting specific areas in hippocampal, amygdala, and basal ganglia regions in SOs that differentiated Global and non-Global SOs during selectively S2.

### Classification of Global and non-Global slow oscillations within and across participants

Once our dataset of labeled matrix representation of depth profiles was prepared, we reasoned that if Global and non-Global SOs were structurally different, multiple classifiers would be able to distinguish between them with high performance. In looking for an analytical framework that introduced the least amount of bias, we sourced 30 classification algorithms using Classification Learner in Matlab (TheMathworks, R2021a), as we appreciated the combination of methods made available in the application, and that each methodology was articulated at different degrees of refinement. We trained each classifier on each individual separately, to study how classifier performance varied across individuals and across algorithms. The rationale for training separate classifiers for each individual is driven by our goal to learn about differences in Global/non-Global SOs in a data-driven way. A crucial conceptual step in leveraging machine learning to compare Global and non-Global SOs is to train and assess the performance of the classifiers in a modality that preserves the true natural variation in the dataset (i.e., enough datapoints are sampled from an appropriately constructed population sample) and minimizes the correlative information that does not drive datapoint labeling but can nonetheless bias classifier performance. In our case, this is especially pertinent when considering that sub-groups of SOs were accrued from the same individual in the same night, hence introducing the potential for the classifiers to be biased in their performance by potential redundancies in the data introduced by sourcing many SOs from each individual. To establish performance, we leverage the Matthews Correlation Coefficient (MCC), which is highly informative for binary classification (Chicco et al., 2021a; Chicco et al., 2021b) and can account for the imbalance in the Global and non-Global SOs found within each individual (details in Methods). We reasoned that if Global and non-Global SOs had intrinsically different depth profiles as encoded in our 51-dimensional approach, then multiple classifiers would show high accuracy across individuals. We also reasoned that if these differences were nonlinear and subtle, then not all classifiers would show high accuracy, and hence all sub-datasets organized by individuals would show a range of variance in the accuracy achieved by the 30 potential classifiers employed. Furthermore, in principle, it was possible that some individuals could have Global and non-Global SOs with highly similar depth profiles, in which case all algorithms would show poor accuracy when trained on data from those individuals.

**FIGURE 4:**
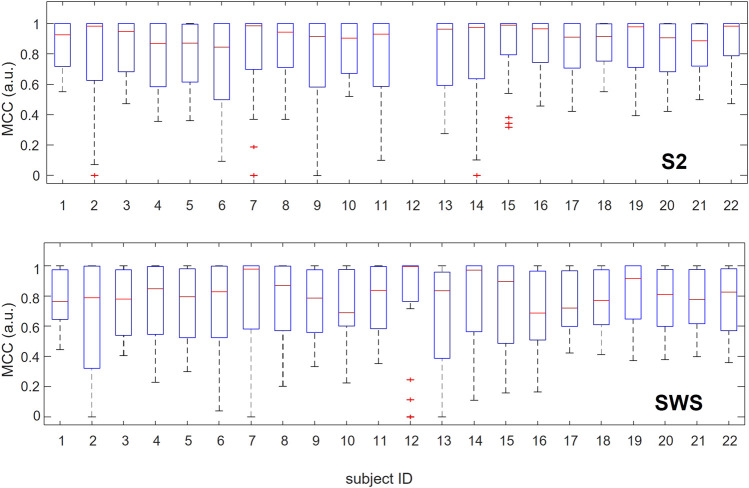
Range of performance for multiple classification algorithms when applied to individual participants separately. For each participant, we report in boxplots the values of performance estimated with MCC and assessed with cross-validation for the 30 machine learning algorithms we tested. The red line marks the median, blue rectangles show the quartile span (25%–75%) and dashed lines the maximum and minimum range. Red crosses mark outliers. Classification of SOs from S2 is reported in the top plot, and SWS in the bottom plot. Participants are listed along the *x*-axis in no particular order, but their identifier (ID) is consistent across plots and tables across the manuscript. Note that participant ^#^12 0 Global SOs detected during S2 sleep, which prevented any classification algorithms for being applicable to this participant in S2. As the full range of values for MCC spans (−1,1), it is notable that for all participants where individual classification was possible (except ^#^12 in S2) the median performance across classification algorithms is above 0.6 and in many cases above 0.8.

In Figure 4, we show the range of variation in performance across 30 classification learning algorithms for each individual, considering the SOs found in S2 and SWS separately. The use of boxplot for visualization emphasizes the overall high performance achieved by the classifiers as a group, showing that medians are above 0.8 for all individuals in S2 and above 0.6 for all individuals in SWS (MCC values range between −1 and 1), but also that performance varies broadly across algorithms. This shows that predictive high-quality differentiation of Global and non-Global SOs is achievable in all individuals in our dataset (except for participant 12, who had no Global SOs detected during their S2 sleep, see Supplementary Table S2). We interpret this as suggesting that while there is a robust differentiation of Global and non-Global SOs in their space-time depth profiles, this differentiation is non-trivial.

**FIGURE 5:**
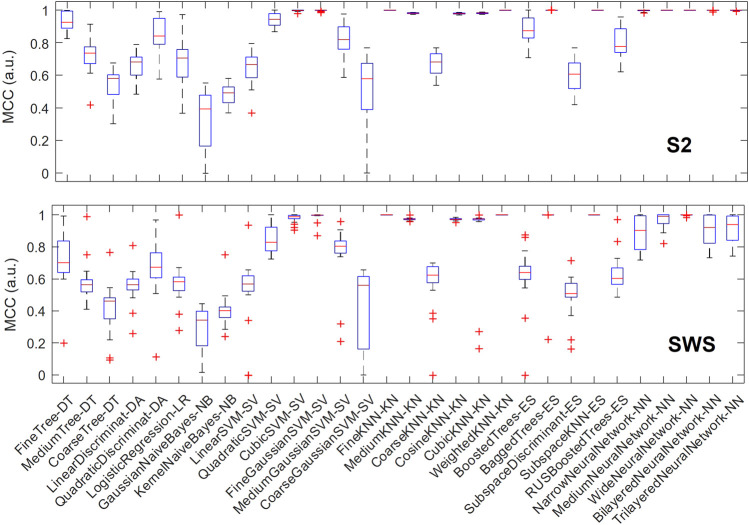
Multiple classification algorithms achieve high MCC performance for all individuals. For each machine learning algorithm that we trained and tested separately in each individual, we report performance (measured with MCC) showing its range across all participants with boxplots. Each boxplot marks the median performance across participants with a red bar, the 25-to-75 percentile range with a blue box, the maximum and minimum value with T-shaped edges and outliers with +-shaped marks. The top plot refers to SOs found in S2 sleep, bottom plot to SWS. Note how multiple algorithms achieve high performance across all individuals and in both sleep stages, supporting the concept that depth profiles of Global and non-Global SOs are measurably different within and across individuals.

We then hypothesized that some classification algorithms could achieve high performance in most or all individuals, and to identify these top performers we plotted the variation in performance for each algorithm across all individuals. As shown in Figure 5, in S2, a number of classification algorithms returned the highest performance for all participants: fine k-nearest neighbors, weighted k-nearest neighbors, ensemble subspace of k-nearest neighbors, and two types of neural networks (medium and wide). Other algorithms also performed near-perfectly for all participants, including bagged trees and some neural networks (narrow, bi-layered and tri-layered). In SWS, the three nearest-neighbor based algorithms that performed highly in S2 showed again optimal performance, while many of the neural-network based algorithms that achieved optimal or close-to-optimal performance in S2 had a slight decline, showing more variance in their performance outcome across individuals (with the wide neural network still performing close to optimal).

The fact that nearest-neighbor-based algorithms had the largest success in distinguishing Global and non-Global SOs across all individuals separately suggests that some structural characteristics of the CSD representation could be strongly contributing to classification performance, in other words, that there would be a subset of interpretable features in the data which were most influential in classification performance. Thus, we reasoned, feature ranking followed by feature selection could reveal region-by-time currents in which Global and non-Global SOs were most different, adding information to what we learn from direct one-to-one comparisons that we performed in Figure 3.

### Feature selection

We have demonstrated high classification and low inter-individual variance across multiple classifiers, suggesting strong support for structural differences between Global and non-Global SOs. Next, we determined the elements in the depth profiles that were most differentiated in Global/non-Global SOs, and thus more likely to be relevant for these classifiers to achieve accuracy. Of note, we have already shown that some features (region-by-time values) has statistically different average values in Global and non-Global SOs across individuals (Figure 3), and that a stronger differentiation among features was found in the time bin around the trough of the SO with this initial approach. Conceptually, however, this approach focuses on the average CSD value assumed by each feature in the Global vs. non-Global category, and hence relies on 44 values (two per individual) to assess differentiation. The classification algorithms were trained and tested on the complete set of all SO values, rather than only their mean, and hence we reason that while having established average differentiation was a necessary and relevant indicator that we should find differentiation of values in Global and non-Global SOs for features, especially those with times around the trough, the actual degree of value differentiation across individual features was best understood with direct quantification on the complete set of all SO values (i.e., capturing variances and not just means of the values).

**FIGURE 6:**
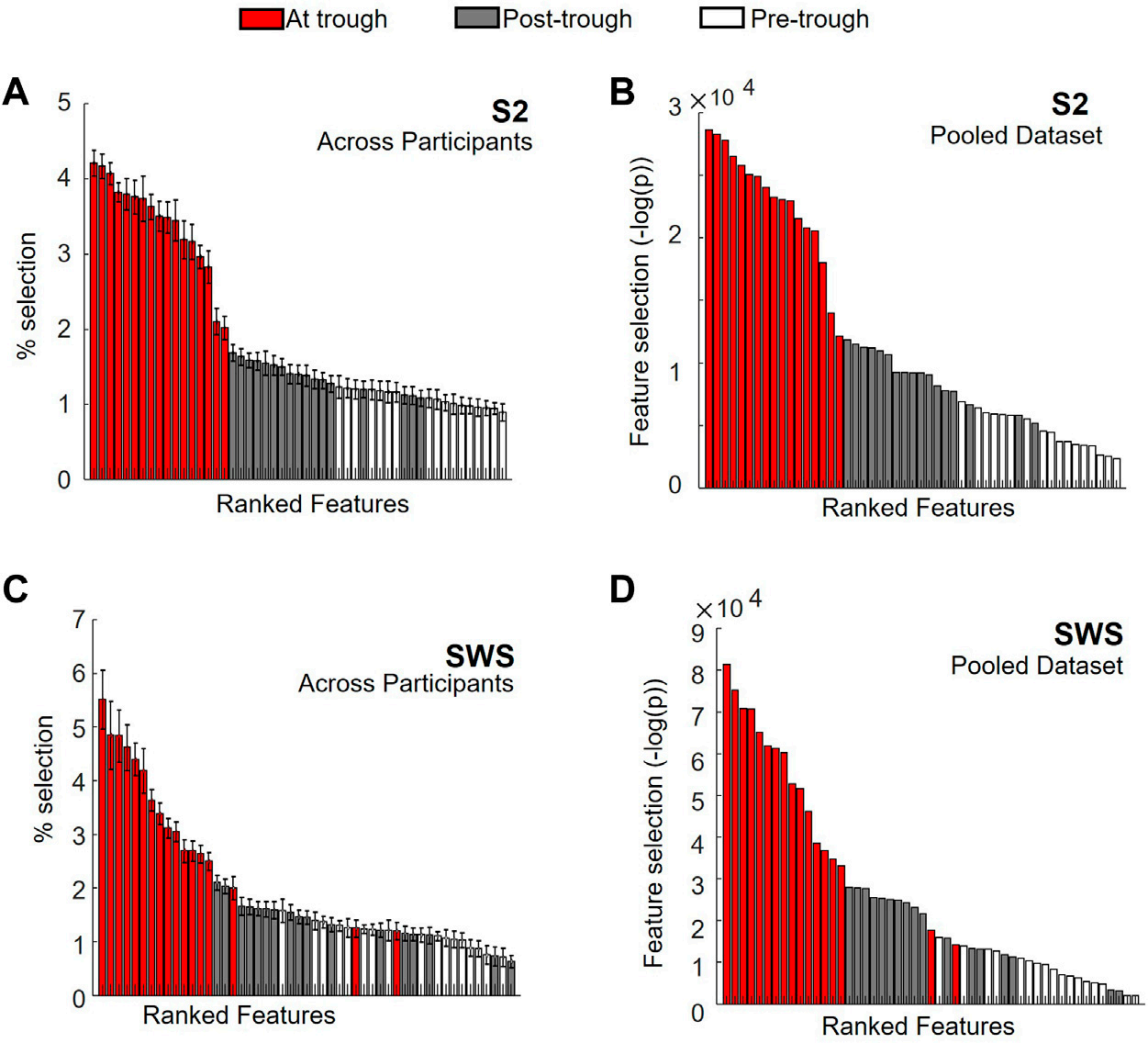
Feature ranking identifies region-time CSD components with largest differentiation in Global vs. non-Global SOs at times near the SO trough. **(A)** Normalized values for feature selection in SOs found during S2, averaged across normalized values found for individual participants separately. Features are sorted according to resulting rank, for ease of reading. Each feature is identified by its region-time pair, where time range is marked with color code, with pre-trough times in white, about-trough time in red and post-trough time in grey (see legend on top). Error bars mark s. e.m. An expanded version of this plot with each feature labeled in detail is reported in Supplementary Figure S4. **(B)** Feature selection for SOs in S2 pooled across all participants, estimated with chi-square (*&KHgr;* ^*2*^) and reported as -log of the *p*-value (i.e., largest values indicate more differentiation for the feature in Global vs. non-Global SOs). Values are sorted from highest to lowest, for ease of reading. Color code is analogous to panel A. An expanded version of this plot with each feature labeled in detail is reported in Supplementary Figure S4. **(C)** Same as A, but for SOs in SWS. An expanded version of this plot with each feature labeled in detail is reported in Supplementary Figure S5. **(D)** Same as in B, but for SOs in SWS. An expanded version of this plot with each feature labeled in detail is reported in Supplementary Figure S5.

To this end, we used univariate feature selection, which in our case was highly interpretable since features in our dataset were region-in-time CSD values. Feature selection was obtained using chi-square and reported using the negative of the logarithms of its *p*-value. This produces a metric that increases with the likelihood that Global SOs and non-Global SOs assume statistically different values in a given feature, and we report this metric for each region-by-time feature in SOs found in S2 or SWS in Figure 6 (and show more details of the same representations in Supplementary Figures S3–S5). Of note, since we are interested in considering the SO differentiation both within and across individuals, we chose to conduct two parallel feature ranking analyses, one separating the analysis in each individual and one pooling all SOs across individuals (in both cases keeping S2 and SWS separate, as we had already seen in Figures 3, 4, 5 that there were slight differences in Global/non-Global differentiation in the two stages). Feature selection with chi square, reported with the negative of the logarithm of its *p*-values, was computed for each individual. The overall magnitude of these values depends on the number of points in each dataset, which can vary considerably across individuals (examples in supporting Supplementary Figure S3). Thus, to integrate the information derived from these individual quantifiers in an average estimator, we normalized each plot by dividing its *y*-axis values by the total sum of values in the whole plot (to force a total area of 1). This results in a scaled feature ranking value, adjusted for the total number of SO in each set, which can then be used in calculating the average value for each feature across individuals. The average feature selection values that we obtained with this approach are shown, sorted by ranking, in Figure 6 (panel A for S2, C for SWS, and Supplementary Figures S4, S5 for more details). The feature selection values obtained by pooling SOs for all individuals in one large dataset are also showed in Figure 6 (panels B for S2 and D for SWS, and Supplementary Figures S4, S5 for more details). In both representations, we color-coded each feature based on its time bin, with white bars for the pre-trough epoch, red bars for the trough epoch, and grey bars for post-trough. As Figure 6 shows, feature selection revealed a strong dominance of currents at times around the SO trough in differentiating Global and non-Global SOs, and this was true for both the individual and pooled data approach, and in both sleep stages.

Our feature selection results contained information about regions as well as time epochs. Within the highly ranked features with currents in the time epoch around the trough, we were interested in establishing which regions held currents that was ranked most selective. Rather than picking an arbitrary number of top features, we decided to use the shape of the ranked histogram to determine the cutoff value. For each *y*-axis value of the histogram, starting with the leftmost bar (the highest ranked) we calculated the gap in feature selection value to the next bar in the ranked order. This differential would peak wherever a large drop was separating one feature from the next (Supplementary Figure S6 shows these gap values for Figures 6A,B). For SOs in S2, we found that the cutoff emerged after the top 15 features, both in the individual and in the pooled dataset. We then compared which regions (all the trough time epochs) were part of this group in each condition (individual or pooled) and found that the same regions were highly ranked in both cases (listed in Table 1), with a high agreement in ranking for top selective features between the individual and the pooled approach (Supplementary Figure S6). For SWS, this approach produced a picture that was less clear-cut, with multiple high peaks in the differential found in the highly ranked features, although again there was a strong ranking agreement between the individual and the pooled dataset analyses (Supplementary Figure S7). We resolved to choose two different cutoffs for the individual (6 features) and the pooled (11 features) histogram, and again compared which regions were found to be most selective between Global and non-Global SOs (Table 1).

**TABLE 1:** Feature selection outcomes.

S2		SWS	
Across Individuals	Pooled Dataset	Across Individuals	Pooled Dataset
Accumbens L Tr.	Accumbens L Tr.	Caudate L Tr.	Caudate L Tr.
Accumbens R Tr.	Accumbens R Tr.	Cortex R Tr.	Caudate R Tr.
Amygdala L Tr.	Amygdala L Tr.	Cortex L Tr.	Brainstem Tr.
Amygdala R Tr.	Caudate L Tr.	Caudate R Tr.	Cortex L Tr.
Caudate L Tr.	Amygdala R T.r	Brainstem Tr.	Cortex R Tr.
Hippocampus R Tr.	Brainstem Tr.	Thalamus R Tr.	Thalamus R Tr.
Cortex R Tr.	Hippocampus R Tr.		Accumbens L Tr.
Brainstem Tr.	Caudate R Tr.		Accumbens R Tr.
Hippocampus L Tr.	Pallidum R T.r		Amygdala L Tr.
Caudate R Tr.	Cortex R Tr.		Amygdala R Tr.
Pallidum R Tr.	Hippocampus L Tr.		Putamen L Tr.
Cortex L Tr.	Pallidum L Tr.		
Pallidum L Tr.	Putamen L Tr.		
Putamen L Tr.	Cortex L Tr.		
Thalamus R Tr.	Thalamus R Tr.		

The abbreviation “Tr.” marks a feature in the trough time bin. Features are sorted according to their ranking emerging from feature selection, and each list is confined to features ranked above the cutoff determined for their feature selection histogram.

As can be seen in Table 1, all regions that were strongly differentiating between Global and non-Global SOs in SWS were also highly ranked in S2. These included the neocortex, the thalamus, the caudate nucleus, and the brainstem. When using the less-stringent cutoff of 11 regions for SOs in SWS, this added currents in the amygdala, nucleus accumbens, and putamen among the most discriminating. In S2, currents in almost all regions (for time epochs around the SO trough) were highly differentiating in Global and non-Global SOs. Beyond regions found to be discriminating in SWS, current in the hippocampus and pallidum were also relevant to differentiation. To evaluate if the features that were highly ranked by selection had enough information on differentiating Global and non-Global SO dynamics to support accurate classification, we compared the performance of classifiers deployed on datapoints there were represented with the full 51 features or the reduced set of highly ranked features. We conducted these test in the pooled datasets only, in S2 testing reduction to the top 15 features (since the same set of 15 features was selected in the individual and pooled dataset) and in SWS we tested both reducing to the top 6 and top 11 features, as indicated in Table 1. We classified Global and non-Global SOs again with all 30 ML algorithms that we had used in the individual sets, and compared performance when training and testing used the full 51 features or the top features only. Of note, these are strong reductions in dimensionality of the space, so we did not necessarily expect maintenance of excellent or perfect performance when the dimensionality reduction was applied. Nonetheless, we hypothesize that the algorithms that performed really well in individual sets would still perform highly in the pooled dataset, and that the reduction in dimensionality driven by feature selection would not damage performance in these algorithms.

**FIGURE 7:**
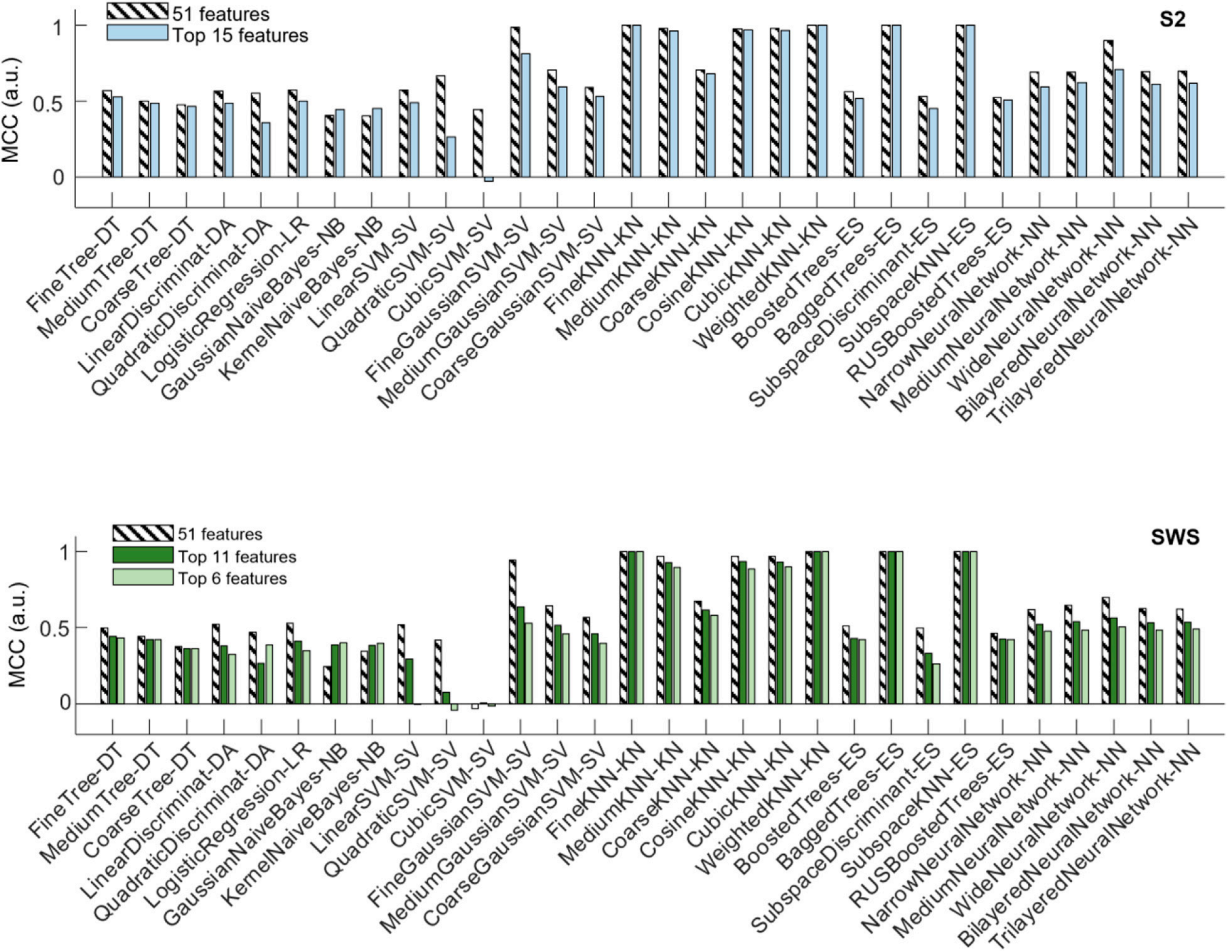
Changes in classifier performance when the depth profiles are reduced to the highest-ranking features. Classifier performance (assessed with MCC) measured with holdout (25%), when training the algorithms with pooled SOs from all participants, SOs from S2 in the top plot, SOs from SWS in the bottom plot. For each algorithm, compare its performance when trained on the dataset encoded by all region-by-time values (51 features, striped bars) to the performance achieved when trained on the dataset encoded only by the top selective features identified in Figure 6 and Table 1 (15 features in S2, and either 11 or 6 features in SWS, full-color bars).

The top panel of Figure 7 shows that a number of classification algorithms reached really high performance (MCC>0.975) in the pooled S2 dataset using all 51 features (represented with striped bars). Those included algorithms that performed highly in the individual datasets (fine, medium, cosine, cubic and weighted KNN, bagged trees and subspace ensemble of KNN). However, not all algorithms that showed high performance in the individual datasets reached comparable performance in the pooled dataset, with multiple neural-network-based algorithms showing less performance in the pooled dataset. One SVM (fine Gaussian) also showed high performance. When reducing features to the top-ranked 15 ones, the selected features were identical in the two selections (from individual feature selection or from pooled, see Table 1). Hence, the top panel of Figure 7 only shows one comparison bar for each algorithm. One can see that all algorithms with excellent performance at 51 features still performed very highly (MCC above 0.96) in the reduced feature environment, with no loss of performance in fine and weighted KNN and in the subspace ensemble KNN, with the exception of the fine Gaussian SVM algorithm, which showed a strong performance loss with dimensionality reduction.

The bottom panel of Figure 7 shows the performance of classification algorithms applied to the pooled SWS dataset. Full 51 features results are shown in striped bars, and one can quickly notice that algorithms that performed highly in S2 also reached high performance in SWS data, again supporting the idea that differentiation of Global and non-Global SO is a relevant phenomenon in both stages, with some structural consistence across NREM sleep. The reduction to top ranked features in the SWS case required the algorithms to confine their information to a much more stringent set of regions (all in the epoch nearby the SO trough), either 11 or 6 features, depending on whether we choose to base our feature selection on pooled or individual feature selection outcomes. Once again, the reduction of dimensionality did not severely affect performance of these algorithms, except for the fine Gaussian SVM. However, there was a small loss in performance, progressively larger as the number of features decreased from 11 to 6, in some of the KNN-based algorithms. Best performing algorithms in all reduced features were fine, medium, weighted and subspace ensemble KNN, and bagged trees.

This dominance in performance of nearest-neighbor-based algorithms, and their performance stability with respect to feature selection suggests that the differentiation of S2 Global and non-Global SOs in their cortical-subcortical estimated currents is most pronounced for epochs near the trough and can be captured by consistent dissimilarities in the current density not just in the neocortex, but also in many subcortical regions. For both SWS and S2, these regions include the thalamus, the brainstem, and the caudate nucleus (in the striatum), in S2 ulterior regions include hippocampus, amygdala and more basal ganglia regions (pallidum, putamen and nucleus accumbens).

## Discussion

We investigated structural differences between Global and non-Global SOs using machine learning to classify depth profiles of SOs across cortical and subcortical regions. To this end, we constructed a methodology that leverages source localization to encode scalp EEG SOs in a four-dimensional (voxel-by-instant) framework and extracts SOs as datapoints in a region-by-time embedding. We then leveraged this framework to analyze differences in Global and non-Global SO profiles, first with basic statistics and then with machine-learning based approaches. We found consistent differences between Global and non-Global SO depth profiles, within and across individuals and across multiple classifiers, with the best performing algorithms relying on k-nearest-neighbors (KNN) principles. We also found that feature selectivity and basic statistics both support that Global and non-Global SOs differentiate depth profiles in SWS and S2. Specifically, differentiating Global and non-Global SOs during both SWS and S2 leverages currents in the cortical, thalamic, and caudate network, and also currents in the brainstem. Furthermore, differentiation in S2 also relies on currents from the amygdala, hippocampus, and multiple basal ganglia regions, which are not involved in differentiation of Global and non-Global SOs in SWS.

There are a number of functional observations that can be relevant to understand the potential significance of these networks of activity differential involvement in Global and non-Global SO. We suggest that our findings have functional implications on two separate levels: 1) the relevance of currents in some specific regions (cortex, thalamus, caudate nucleus, and brainstem) to distinguish Global and non-Global SOs in both S2 and SWS, and 2) the fact that differentiation in S2 relies on a larger and more generalized network of cortical-subcortical activity than in SWS, involving additional regions like hippocampus, amygdala and many basal ganglia areas.

Our results show that the pillars of Global/non-Global SO differentiation, regardless of sleep stage are cortex, thalamus, caudate nucleus, and brainstem. The emergence and propagation of sleep spindles in the EEG relies on cortico-thalamic bidirectional interaction, and sleep spindles have a preferential coordination with Global SOs (Malerba et al., 2019), so the relevance of thalamic and cortical currents could in part be related to this differentiation in interaction with spindles. Caudate nucleus activity is connected to associative and procedural learning, working memory, executive function and is important to motor function (Driscoll et al., 2022), with the network connecting currents in the cortex, thalamus and caudate nucleus relevant to the processing of reward (Schultz, 2016) and memories guided by the dorsal striatum (which includes the caudate nucleus) especially processed during SWS (Watts et al., 2012). Since reward during wake influences what aspects of learned experiences will gain salience, it can in turn influence sleep-mediated information processing, possibly reflected in a differentiation of Global and non-Global SO dynamics in the same network. Finally, the relevance of brainstem currents to differentiation of Global and non-Global SO dynamics could be connected to known autonomic-central events (ACE) found to coordinate changes in heart rate dynamics with temporary increases in SO and spindle emergence (Naji et al., 2019; Chen et al., 2020). The brainstem includes nuclei that are involved in modulating SWS depth (Anaclet and Fuller, 2017) and heart rate deceleration (Monge Argilés et al., 2000), and could be the central hub orchestrating fluctuations in both heart rate modulation and SO and spindle emergence (Chen et al., 2020). While at this stage it is not clear if there is a differential involvement of Global and non-Global SOs in ACEs, our data showing that brainstem currents strongly differentiate between the two SO types in both NREM sleep stages suggest that this could be the case.

The other surprising finding from our analysis is that differentiation of Global and non-Global SO relies on an extensive network of activity during S2, larger than the network that emerged for SWS, including activity in the hippocampus, amygdala and many basal ganglia regions. The relevance of currents in the cortex, hippocampus, and thalamus during the SO trough to SO differentiation during S2 is consistent with the large body of research implicating SOs in the coordination of episodic memory reactivation in these regions during sleep (Ji and Wilson, 2007; Mehta, 2007; Chauvette et al., 2012; Dudai et al., 2015; Latchoumane et al., 2017; Klinzing et al., 2019), and with our previous study which specifically identified Global SOs as preferentially connected to this memory network (Niknazar et al., 2022). In a similar fashion, a role for coordination of currents in the amygdala with the episodic memory network in differentiating Global and non-Global SOs could be reflective of a relative abundance of emotional memory processing during S2 (Kaestner et al., 2013). As per the relative involvement of many basal ganglia regions, the caudate nucleus is selectively less active in SWS compared to S2 sleep (Kaufmann et al., 2006), a property which could account for enhanced differentiability of Global/non-Global SO dynamics during SWS. One can infer that possibly the strong differences between S2 and SWS in the number and organization of SOs (SO trains are much more frequent and indeed a hallmark of SWS, compared to S2) result in a non-uniform depth profile differentiation of Global and non-Global SOs in these two sleep stages. This perspective would agree with theories that support complementary mechanistic roles in memory processing of S2 and SWS NREM stages (Genzel et al., 2014) and further underscores the need to carefully choose whether to separate S2 and SWS SO analysis depending on which question a study aims to address.

Mechanistically, it is worth emphasizing that we found a strong differentiation of SO dynamics in Global and non-Global SOs in many brain regions, and with an emphasis for dynamics at times near the trough, despite Global and non-Global SO profiles being identified on a larger time scale (up to 400 ms-long delays). When discussing the functional role of SOs in restorative and cognitive functions of sleep, their role as “coordinators” of activity across brain regions is often invoked, within the known hypothesis that they coordinate a wide network of brain regions during memory reactivation, which in turn can lead to synaptic reorganization and stabilization of hippocampal-dependent memories. This concept is connected to the known property of hierarchical nesting, where faster rhythmic oscillations (spindles and ripples) pick a coordinated timing of occurrence in relation to the phase to an ongoing slower oscillation. Our previous research showed that Global SOs have stronger coordination with spindles compared to other SO types (Figure 7A in Malerba et al., 2019). The potential differentiation of Global and non-Global SOs CSDs in specific brain regions that are important for memory and other cognitive processes (such as reward, and brain-body interaction), implies that perhaps not all SOs are coordinating exactly the same regions every time, but rather that Global SOs could be serving a selective role in coordinating cortical-subcortical activity more strongly than non-Global SOs. Our analysis also shows a dominance of differentiation in times close to the SO trough as opposed to preceding or following, suggesting that if the mechanistic role of ‘activity coordinators’ of the SOs is to be seen in how spindles appear following the SO trough, the specific factors that allow a Global or a non-Global SO to influence these events are most likely found at times close to the trough. This supports the idea of an ‘event cascade’ where the articulation of SO-spindle coordination in space-time is crucially initiated at the time of the trough, and the early stages of emergence toward the Up State influence the overall propagation and activation in the network to a strong degree. This suggests a picture in which the dynamics near the SO trough sets the timing-and-location stage for the network to be ready (a few hundreds of ms later) to process specific cell activity across many regions selectively. One can then speculate that faster dynamics (such as spindle activity) nested within these established coordination patterns could be the substrate for the more specific synaptic changes.

We have introduced an explicit methodology to leverage depth profiles built on source localization to identify dynamical differences in rhythms captured in the scalp EEG. The usefulness of this approach is built on our ability to interpret (at least to some degree) the outcome of the classifiers we deployed, which is not a guaranteed feature since explainability of machine learning and artificial intelligence is an ongoing open field (Linardatos et al., 2020; Belle and Papantonis, 2021). To maximize interpretability, we built a framework tailored to our research question, by first encoding our datapoints in appropriate matrices - so that features could be explicitly connected to meaningful objects in our analysis - sand then deploying a collection of classifiers on a person-by-person base, and define as preferable the outcome of classification algorithms that could reach consistent high accuracy for all participants when individually trained. The choice of data encoding methodology is crucial to the interpretability of our results in terms of activation of cortical-subcortical regions at times important for SO occurrence and interaction with other brain rhythms, such as spindles. The choice of selecting algorithms on inter-individual accuracy allows us to estimate the differentiability of Global and non-Global depth profiles while taking into account that during a night of sleep, brain dynamics is highly more similar within, rather than across, individuals. This structured methodology can be expanded to other oscillations that show differential scalp profiles (Malerba et al., 2020), and represents a novel principled approach to evaluate within-individual differentiability of sleep oscillations based on their space-time profiles. This is a crucial step in understanding the mechanistic connection between observed rhythms and their functional roles, as potential changes in network connectivity or cells properties driven by the presence and coordination of these rhythms in the brain can only be experimentally assessed with localized approaches.

In the current study, we did not separate the non-Global SO category in Frontal and Local SOs, despite our original analysis showing that these non-Global SOs are different (Malerba et al., 2019). This is driven by our current goal, which is to identify if there are specific Global SOs properties in their depth profiles that make them uniquely relevant to sleep-dependent memory processing, and can possibly in the future be used to distinguish Global SOs from non-Global ones in real time. However, all steps of our approach can be applied to any finite number of SO types, and hence follow up studies can expand on our current perspective to include classifications across the three SO types.

Our current study has some technical limitations. As is known, inverse problems are intrinsically ill-posed insofar as the number of inputs (EEG value at channel locations) is much fewer than the number of outputs (CSD values at all voxels). Our analysis also limits us to passive estimates based on conductivity and electric fields, lacking the “active” component of how cell activity (spiking, synaptic currents, nonlinear membrane currents) contributes to emergence of Global and non-Global SOs in the brain. In ideal circumstances, one would be able to record with the time resolution of EEG from multiple cortical and subcortical regions during natural sleep. Currently, non-invasive methods that allow depth recording of brain dynamics have much poorer time resolution than EEG, and because we are interested in oscillatory dynamics in relation to function, a high time resolution is crucial. This led us to the choice of estimating depth dynamics from EEG data rather than try and acquire it with other methods. However, future studies that combine EEG and in-depth recording of sleep dynamics could further test our estimates of strong differences in depth profiles of Global and non-Global SOs. Because of the spatially limited nature of depth recording in the human brain, one could only compare estimate predictions to actual recordings within such spatial bounds. However, eventual discrepancies between depth data and EEG-drive predictions could be leveraged to improve estimate models, by extrapolating potential generalizability to other, anatomically similar, regions.

We relied on a well-established standard head model (the MNI package) as a simplification of the real complexity of a study of sleep brain dynamics in a population given our data acquisition did not include MRI. Future studies that include MRI acquisition for computational estimates would improve and further test the validity of our conclusions. Of note, due to the semi-stationary nature of the biological properties assessed with MRI, a study of this type would be successful as long as the time delay between MRI and sleep polysomnography acquisition was limited to a few months. We also chose to model a relatively limited number of subcortical structures and consider all neocortical regions as one (separated only in left and right). A more refined differentiation of cortical regions and a more extensive assessment of subcortical regions could expand the range of functional interpretations of our analysis outcome, possibly to frontal or lateralized sleep-dependent information processing, as sleep oscillations are known to organize in space coherently with awake encoding behavior (Huber et al., 2004; Piantoni et al., 2015; Bernardi et al., 2019). This expansion could of course be numerically achieved in follow up studies utilizing MRI acquisition, as this would allow for personalized choices on the CSD reconstruction for each individual. Such framework could extend the relevance of our approach to populations that have sleep impairments that may be related to SO dynamics (e.g., Hypersomniacs (Plante et al., 2012)).

## Data Availability

The data analyzed in this study is subject to the following licenses/restrictions: While the entire sleep dataset is not publicly available, specific quantifiers can be shared upon request to the corresponding author. Requests to access these datasets should be directed to malerba.2@osu.edu.

## References

[B51] Anaclet C. Fuller P. M. (2017). Brainstem regulation of slow-wave-sleep. Curr. Opin. Neurobiol. 44, 139–143. 10.1016/j.conb.2017.04.004 28500870 PMC5607774

[B1] Bazhenov M. Timofeev I. Steriade M. Sejnowski T. J. (2002). Model of thalamocortical slow-wave sleep oscillations and transitions to activated States. J. Neurosci. 22, 8691–8704. 10.1523/jneurosci.22-19-08691.2002 12351744 PMC6757797

[B2] Belle V. Papantonis I. (2021). Principles and practice of explainable machine learning. Front. Big Data 4, 688969. 10.3389/fdata.2021.688969 34278297 PMC8281957

[B3] Bernardi G. Betta M. Cataldi J. Leo A. Haba-Rubio J. Heinzer R. (2019). Visual imagery and visual perception induce similar changes in occipital slow waves of sleep. J. Neurophysiol. 121, 2140–2152. 10.1152/jn.00085.2019 30943100

[B4] Chauvette S. Seigneur J. Timofeev I. (2012). Sleep oscillations in the thalamocortical system induce long-term neuronal plasticity. Neuron 75, 1105–1113. 10.1016/j.neuron.2012.08.034 22998877 PMC3458311

[B5] Chen P. C. Whitehurst L. N. Naji M. Mednick S. C. (2020). Autonomic/central coupling benefits working memory in healthy young adults. Neurobiol. Learn. Mem. 173, 107267. 10.1016/j.nlm.2020.107267 32535198

[B6] Chicco D. Totsch N. Jurman G. (2021a). The Matthews correlation coefficient (MCC) is more reliable than balanced accuracy, bookmaker informedness, and markedness in two-class confusion matrix evaluation. BioData Min. 14, 13. 10.1186/s13040-021-00244-z 33541410 PMC7863449

[B7] Chicco D. Warrens M. J. Jurman G. (2021b). The Matthews correlation coefficient (MCC) is more informative than cohen’s kappa and brier score in binary classification assessment. IEEE Access 9, 78368–78381. 10.1109/access.2021.3084050

[B8] Dang-Vu T. T. Schabus M. Desseilles M. Albouy G. Boly M. Darsaud A. (2008). Spontaneous neural activity during human slow wave sleep. Proc. Natl. Acad. Sci. U. S. A. 105, 15160–15165. 10.1073/pnas.0801819105 18815373 PMC2567508

[B9] Diekelmann S. Born J. (2010). The memory function of sleep. Nat. Rev. Neurosci. 11, 114–126. 10.1038/nrn2762 20046194

[B10] Driscoll M. E. Bollu P. C. Tadi P. (2022). Neuroanatomy, nucleus caudate. Treasure Island (FL): StatPearls. 32491339

[B11] Dudai Y. Karni A. Born J. (2015). The consolidation and transformation of memory. Neuron 88, 20–32. 10.1016/j.neuron.2015.09.004 26447570

[B12] Fultz N. E. Bonmassar G. Setsompop K. Stickgold R. A. Rosen B. R. Polimeni J. R. (2019). Coupled electrophysiological, hemodynamic, and cerebrospinal fluid oscillations in human sleep. Science 366, 628–631. 10.1126/science.aax5440 31672896 PMC7309589

[B13] Genzel L. Kroes M. C. Dresler M. Battaglia F. P. (2014). Light sleep versus slow wave sleep in memory consolidation: A question of global versus local processes? Trends Neurosci. 37, 10–19. 10.1016/j.tins.2013.10.002 24210928

[B14] Gramfort A. Papadopoulo T. Olivi E. Clerc M. (2010). OpenMEEG: Opensource software for quasistatic bioelectromagnetics. Biomed. Eng. Online 9, 45. 10.1186/1475-925X-9-45 20819204 PMC2949879

[B15] Huber R. Ghilardi M. F. Massimini M. Tononi G. (2004). Local sleep and learning. Nature 430, 78–81. 10.1038/nature02663 15184907

[B16] Ji D. Wilson M. A. (2007). Coordinated memory replay in the visual cortex and hippocampus during sleep. Nat. Neurosci. 10, 100–107. 10.1038/nn1825 17173043

[B17] Kaestner E. J. Wixted J. T. Mednick S. C. (2013). Pharmacologically increasing sleep spindles enhances recognition for negative and high-arousal memories. J. Cogn. Neurosci. 25, 1597–1610. 10.1162/jocn_a_00433 23767926

[B18] Kaufmann C. Wehrle R. Wetter T. C. Holsboer F. Auer D. P. Pollmacher T. (2006). Brain activation and hypothalamic functional connectivity during human non-rapid eye movement sleep: An EEG/fMRI study. Brain 129, 655–667. 10.1093/brain/awh686 16339798

[B19] Klinzing J. G. Niethard N. Born J. (2019). Mechanisms of systems memory consolidation during sleep. Nat. Neurosci. 22, 1598–1610. 10.1038/s41593-019-0467-3 31451802

[B20] Kybic J. Clerc M. Abboud T. Faugeras O. Keriven R. Papadopoulo T. (2005). A common formalism for the integral formulations of the forward EEG problem. IEEE Trans. Med. Imaging 24, 12–28. 10.1109/tmi.2004.837363 15638183

[B21] Ladenbauer J. Kulzow N. Passmann S. Antonenko D. Grittner U. Tamm S. (2016). Brain stimulation during an afternoon nap boosts slow oscillatory activity and memory consolidation in older adults. Neuroimage 142, 311–323. 10.1016/j.neuroimage.2016.06.057 27381076

[B22] Latchoumane C. V. Ngo H. V. Born J. Shin H. S. (2017). Thalamic spindles promote memory formation during sleep through triple phase-locking of cortical, thalamic, and hippocampal rhythms. Neuron 95, 424–435. 10.1016/j.neuron.2017.06.025 28689981

[B23] Lee H. Xie L. Yu M. Kang H. Feng T. Deane R. (2015). The effect of body posture on brain glymphatic transport. J. Neurosci. 35, 11034–11044. 10.1523/JNEUROSCI.1625-15.2015 26245965 PMC4524974

[B24] Linardatos P. Papastefanopoulos V. Kotsiantis S. (2020). Explainable AI: A Review of Machine Learning Interpretability Methods. Entropy (Basel) 23, 18. 10.3390/e23010018 33375658 PMC7824368

[B25] Malerba P. Whitehurst L. N. Simons S. B. Mednick S. C. (2019). Spatio-temporal structure of sleep slow oscillations on the electrode manifold and its relation to spindles. Sleep 42. 10.1093/sleep/zsy197 PMC6335956 30335179

[B26] Malerba P. Whitehurst L. Mednick S. (2020). Topographical analysis of sleep spindles and their coordination with slow oscillations. Sleep. Phila. 43, A37–A38. 10.1093/sleep/zsaa056.091 PMC9366646 35666552

[B27] Maquet P. (2001). The role of sleep in learning and memory. Science 294, 1048–1052. 10.1126/science.1062856 11691982

[B28] Marshall L. Helgadottir H. Molle M. Born J. (2006). Boosting slow oscillations during sleep potentiates memory. Nature 444, 610–613. 10.1038/nature05278 17086200

[B29] Massimini M. Huber R. Ferrarelli F. Hill S. Tononi G. (2004). The sleep slow oscillation as a traveling wave. J. Neurosci. 24, 6862–6870. 10.1523/JNEUROSCI.1318-04.2004 15295020 PMC6729597

[B30] Mehta M. R. (2007). Cortico-hippocampal interaction during up-down states and memory consolidation. Nat. Neurosci. 10, 13–15. 10.1038/nn0107-13 17189946

[B31] Mikutta C. Feige B. Maier J. G. Hertenstein E. Holz J. Riemann D. (2019). Phase-amplitude coupling of sleep slow oscillatory and spindle activity correlates with overnight memory consolidation. J. Sleep. Res. 28, e12835. 10.1111/jsr.12835 30848042

[B32] Molle M. Born J. (2011). Slow oscillations orchestrating fast oscillations and memory consolidation. Prog. Brain Res. 193, 93–110. 10.1016/B978-0-444-53839-0.00007-7 21854958

[B52] Monge Argilés J. A. Palacios Ortega F. Vila Sobrino J. A. Bautista Prados J. Pérez Vicente J. A. Morales Ortiz A. (2000). Brainstem lesions decrease heart rate variability. Neurologia. 15 (4), 158–163. 10846883

[B33] Naji M. Krishnan G. P. Mcdevitt E. A. Bazhenov M. Mednick S. C. (2019). Coupling of autonomic and central events during sleep benefits declarative memory consolidation. Neurobiol. Learn. Mem. 157, 139–150. 10.1016/j.nlm.2018.12.008 30562589 PMC6425961

[B34] Ngo H. V. Martinetz T. Born J. Molle M. (2013). Auditory closed-loop stimulation of the sleep slow oscillation enhances memory. Neuron 78, 545–553. 10.1016/j.neuron.2013.03.006 23583623

[B35] Niknazar H. Malerba P. Mednick S. C. (2022). Slow oscillations promote long-range effective communication: The key for memory consolidation in a broken-down network. Proc. Natl. Acad. Sci. U. S. A. 119, e2122515119. 10.1073/pnas.2122515119 35733258 PMC9245646

[B36] Ong J. L. Lo J. C. Chee N. I. Santostasi G. Paller K. A. Zee P. C. (2016). Effects of phase-locked acoustic stimulation during a nap on EEG spectra and declarative memory consolidation. Sleep. Med. 20, 88–97. 10.1016/j.sleep.2015.10.016 27318231

[B37] Papalambros N. A. Santostasi G. Malkani R. G. Braun R. Weintraub S. Paller K. A. (2017). Acoustic enhancement of sleep slow oscillations and concomitant memory improvement in older adults. Front. Hum. Neurosci. 11, 109. 10.3389/fnhum.2017.00109 28337134 PMC5340797

[B38] Pascual-Marqui R. D. (2002). Standardized low-resolution brain electromagnetic tomography (sLORETA): technical details. Methods Find. Exp. Clin. Pharmacol. 24, 5–12. 12575463

[B39] Piantoni G. Van der Werf Y. D. Jensen O. Van Someren E. J. (2015). Memory traces of long-range coordinated oscillations in the sleeping human brain. Hum. Brain Mapp. 36, 67–84. 10.1002/hbm.22613 25139521 PMC6869021

[B40] Plante D. T. Landsness E. C. Peterson M. J. Goldstein M. R. Wanger T. Guokas J. J. (2012). Altered slow wave activity in major depressive disorder with hypersomnia: A high density EEG pilot study. Psychiatry Res. 201, 240–244. 10.1016/j.pscychresns.2012.03.001 22512951 PMC3361575

[B41] Rasch B. Born J. (2013). About sleep's role in memory. Physiol. Rev. 93, 681–766. 10.1152/physrev.00032.2012 23589831 PMC3768102

[B42] Rechtschaffen A. (1968). A manual for standardized terminology, techniques and scoring system for sleep stages in human subjects. Los Angeles: Brain information service.

[B43] Saletin J. M. (2015). Húmë: Open-source MATLAB sleep scoring toolbox.

[B44] Schultz W. (2016). Reward functions of the basal ganglia. J. Neural Transm. 123, 679–693. 10.1007/s00702-016-1510-0 26838982 PMC5495848

[B45] Tadel F. Baillet S. Mosher J. C. Pantazis D. Leahy R. M. (2011). Brainstorm: A user-friendly application for MEG/EEG analysis. Comput. Intell. Neurosci. 2011, 879716. 10.1155/2011/879716 21584256 PMC3090754

[B46] Timofeev I. Grenier F. Bazhenov M. Sejnowski T. J. Steriade M. (2000). Origin of slow cortical oscillations in deafferented cortical slabs. Cereb. Cortex 10, 1185–1199. 10.1093/cercor/10.12.1185 11073868

[B47] Tononi G. Cirelli C. (2006). Sleep function and synaptic homeostasis. Sleep. Med. Rev. 10, 49–62. 10.1016/j.smrv.2005.05.002 16376591

[B48] Walker M. P. Stickgold R. (2006). Sleep, memory, and plasticity. Annu. Rev. Psychol. 57, 139–166. 10.1146/annurev.psych.56.091103.070307 16318592

[B49] Watts A. Gritton H. J. Sweigart J. Poe G. R. (2012). Antidepressant suppression of non-REM sleep spindles and REM sleep impairs hippocampus-dependent learning while augmenting striatum-dependent learning. J. Neurosci. 32, 13411–13420. 10.1523/JNEUROSCI.0170-12.2012 23015432 PMC3712834

[B50] Wendel K. Vaisanen O. Malmivuo J. Gencer N. G. Vanrumste B. Durka P. (2009). EEG/MEG source imaging: methods, challenges, and open issues. Comput. Intell. Neurosci. 2009, 1–12. 10.1155/2009/656092 PMC2715569 19639045

