# Supplementary material for: Global and non-Global slow oscillations differentiate in their depth profiles
Source: Front Netw Physiol. 2022 Oct 24;2:947618. doi: 10.3389/fnetp.2022.947618 (PMC10013040; doi:10.3389/fnetp.2022.947618)
Supplement: Supplementary file 1 [file DataSheet1.docx]

Global and non-Global Slow Oscillations differentiate in their depth profiles

Supplementary Material

**Seok S.C., McDevitt E., Mednick S.C., Malerba P.**

# Sleep Outcomes Table:

| **Total Sleep Time** | **465.36 ± 7.67** |
| --- | --- |
| **S1** | **22.1591 ± 10.40** |
| **S2** | **199.75 ± 25.92** |
| **SWS** | **126.68 ± 31.61** |
| **REM** | **102.07 ± 14.96** |
| **Sleep Onset** | **14.04 ± 6.71** |
| **WASO** | **5.30 ± 4.75** |

**Table S1. Sleep outcomes for our 22 participants.** WASO: wake after sleep onset. For each value, time is reported in minutes.

# Count of Global and non-Global SO in S2 and SWS

| Participant ID | SOs in S2 | Global SOs in S2 | Non-Global SOs in S2 | SOs in SWS | Global SOs in SWS | Non-Global SOs in SWS |
| --- | --- | --- | --- | --- | --- | --- |
| 1 | 12016 | 6259 | 5757 | 76568 | 35684 | 40884 |
| 2 | 9263 | 1436 | 7827 | 26372 | 2800 | 23572 |
| 3 | 11244 | 3690 | 7554 | 66459 | 17821 | 48638 |
| 4 | 15677 | 6591 | 9086 | 33090 | 4967 | 28123 |
| 5 | 16835 | 7572 | 9263 | 58246 | 16097 | 42149 |
| 6 | 12962 | 3790 | 9172 | 34982 | 7573 | 27409 |
| 7 | 5649 | 884 | 4765 | 8784 | 1468 | 7316 |
| 8 | 10035 | 4751 | 5284 | 13406 | 4136 | 9270 |
| 9 | 13702 | 4526 | 9176 | 49634 | 17210 | 32424 |
| 10 | 15203 | 7244 | 7959 | 56065 | 20443 | 35622 |
| 11 | 10689 | 2934 | 7755 | 26649 | 7098 | 19551 |
| 12 | 2086 | 0 | 2086 | 4164 | 100 | 4064 |
| 13 | 9911 | 2173 | 7738 | 20128 | 2304 | 17824 |
| 14 | 7224 | 1064 | 6160 | 16009 | 1114 | 14895 |
| 15 | 6917 | 554 | 6363 | 18798 | 2774 | 16024 |
| 16 | 10908 | 6055 | 4853 | 44873 | 17759 | 27114 |
| 17 | 14994 | 8447 | 6547 | 110766 | 58393 | 52373 |
| 18 | 16840 | 8868 | 7972 | 77381 | 32620 | 44761 |
| 19 | 8636 | 1676 | 6960 | 18880 | 3727 | 15153 |
| 20 | 15408 | 7624 | 7784 | 46514 | 16624 | 29890 |
| 21 | 17589 | 9563 | 8026 | 84597 | 42546 | 42051 |
| 22 | 7607 | 1727 | 5880 | 28711 | 8877 | 19834 |
| SUM | 251395 | 97428 | 153967 | 921076 | 322135 | 598941 |

**Table S2. Count of SOs per participant, divided by NREM sleep stage.**

# Statistical comparisons for Figure 3 – S2

We use repeated measures ANOVA. Indep Vars: region and SO type Dep variable: CSD (a.u.).

## Pre-trough time bin:

| 'Source' | 'SS' | 'df' | 'MS' | 'F' | 'p' |
| --- | --- | --- | --- | --- | --- |
| 'Region' | 3.43e-2 | 16 | 2.15e-3 | 63.83 | 9.84e-125 |
| 'SO Type' | 2.87e-3 | 1 | 2.87e-3 | 85.36 | 2.95e-19 |
| 'Region x SO Type' | 1.95e-3 | 16 | 1.2e-4 | 3.62 | 2.32e-6 |
| Error | 2.34e-2 | 697 | 3.00e-5 |  |  |
| Total | 6.23e-2 | 730 |  |  |  |

This finds an effect of region, SO type and interaction. Post-hoc: The following regions showed statistically different Global and non-Global CSD: 'AccumbensL' 'AccumbensR' 'AmygdalaL' 'AmygdalaR' 'HippocampusR' 'PallidumL' 'PallidumR'

## Trough time bin:

| 'Source' | 'SS' | 'df' | 'MS' | 'F' | 'p' |
| --- | --- | --- | --- | --- | --- |
| 'Region' | 0.12 | 16 | 7.55e-3 | 105.02 | 2.36e-173 |
| 'SO Type' | 4.32e-2 | 1 | 4.32e-2 | 600.52 | 3.92e-96 |
| 'Region x SO Type' | 0.01 | 16 | 6.70e-4 | 9.32 | 3.53e-21 |
| Error | 5.01e-2 | 697 | 7e-5 |  |  |
| Total | 2.23e-1 | 730 |  |  |  |

This finds an effect of region, SO type and interaction. Post-hoc: All regions showed statistically different Global and non-Global CSD.

## Post-trough time bin:

| 'Source' | 'SS' | 'df' | 'MS' | 'F' | 'p' |
| --- | --- | --- | --- | --- | --- |
| 'Region' | 4.14e-2 | 16 | 2.58e-3 | 67.03 | 4.00e-129 |
| 'SO Type' | 7.24e-3 | 1 | 7.24e-3 | 187.82 | 5.04e-38 |
| 'Region x SO Type' | 2.14e-3 | 16 | 1.30e-4 | 3.47 | 5.40e-06 |
| Error | 2.69e-2 | 697 | 4e-5 |  |  |
| Total | 7.73e-2 | 730 |  |  |  |

This finds an effect of Region, SO type and interaction. Post-hoc: The following regions showed statistically different Global and non-Global CSD:. 'AccumbensL' 'AccumbensR' 'AmygdalaL' 'AmygdalaR' 'HippocampusL' 'HippocampusR' 'PallidumL' 'PallidumR' 'PutamenL'

# Statistical comparisons for Figure 3 – SWS

We use repeated measures ANOVA. Indep Vars: region and SO type Dep variable: CSD (a.u.).

## Pre-trough time bin:

| 'Source' | 'SS' | 'df' | 'MS' | 'F' | 'p' |
| --- | --- | --- | --- | --- | --- |
| 'Region' | 1.39e-2 | 16 | 8.7e-4 | 5.77 | 0 |
| 'SO Type' | 4.7e-4 | 1 | 4.7e-4 | 3.14 | 0.077 |
| 'Region x SO Type' | 1.8e-4 | 16 | 1e-5 | 0.07 | 1 |
| Error | 0.11 | 714 | 1.5e-4 |  |  |
| Total | 0.12 | 747 |  |  |  |

This finds an effect of region, but not SO type and interaction. Post-hoc (redundant): The following regions showed statistically different Global and non-Global CSD: 'AmygdalaR'

## Trough time bin:

| 'Source' | 'SS' | 'df' | 'MS' | 'F' | 'p' |
| --- | --- | --- | --- | --- | --- |
| 'Region' | 7.91e-3 | 16 | 4.9e-4 | 3.25 | 0 |
| 'SO Type' | 0.01 | 1 | 0.01 | 68.82 | 0 |
| 'Region x SO Type' | 7.3e-4 | 16 | 5e-5 | 0.3 | 1 |
| Error | 0.11 | 714 | 1.5e-4 |  |  |
| Total | 0.13 | 747 |  |  |  |

This finds an effect of region, and SO type but not interaction. Post-hoc: The following regions showed statistically different Global and non-Global CSD: 'AccumbensL' 'AccumbensR' 'AmygdalaL' 'AmygdalaR' 'Brainstem' 'CaudateL' 'CaudateR' 'CortexR' 'HippocampusL' 'HippocampusR' 'PutamenL' 'PutamenR' 'ThalamusL' 'ThalamusR'

## Post-trough time bin:

| 'Source' | 'SS' | 'df' | 'MS' | 'F' | 'p' |
| --- | --- | --- | --- | --- | --- |
| 'Region' | 1.22e-2 | 16 | 7.6e-4 | 4.95 | 0 |
| 'SO Type' | 1.53e-3 | 1 | 1.53e-3 | 9.92 | 1.7e-3 |
| 'Region x SO Type' | 2.1e-4 | 16 | 1e-5 | 0.09 | 1 |
| Error | 0.11 | 714 | 1.5e-4 |  |  |
| Total | 0.12 | 747 |  |  |  |

This finds an effect of Region, SO type but not interaction. Post-hoc: The following regions showed statistically different Global and non-Global CSD:. 'AccumbensL' 'AccumbensR' 'AmygdalaL' 'AmygdalaR'

# Figure S1: example distributions of CSD values for one individual – S2


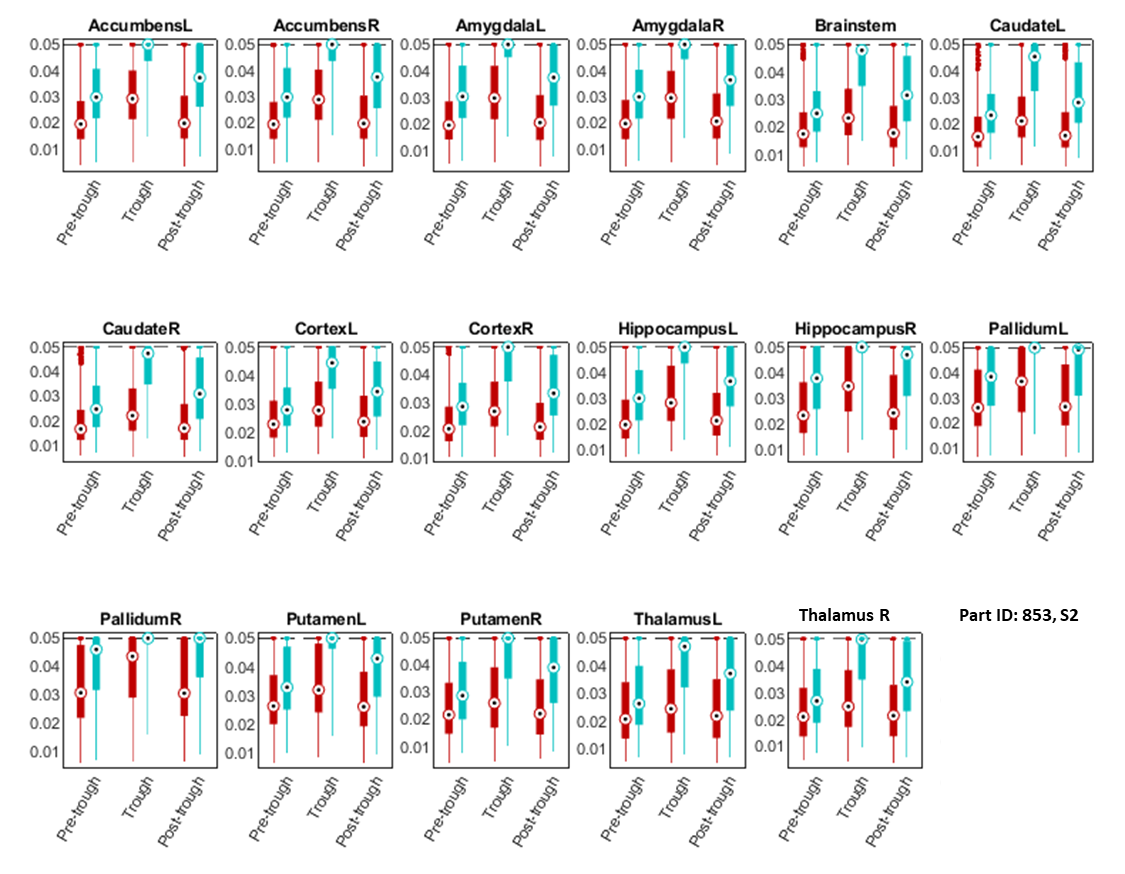


**Fig S1. CSD values for one example participant, separated in Global and non-Global SOs, and by region and time**, **in S2.** In each panel we show the distribution of CSD values for a given region, separated in time bins before, during and after the trough (labeled “Pre-trough”, “Trough” and “Post-trough”). For every plot, the circle marks the median and thick bars mark the quartiles (25% to 75%). In red, we show the CSD values distribution across all the non-Global SOs detected for this specific participant during their Stage 2 sleep (all S2 SOs found across the sleep night considered). In light blue, we have the analogous plots for Global SOs. Note that for needs of uniform visualization, the values shown are truncated at 0.05. CSD is in arbitrary units. Participant ID 835, SOs found during S2.

# Figure S2: example distributions of CSD values for one individual – SWS


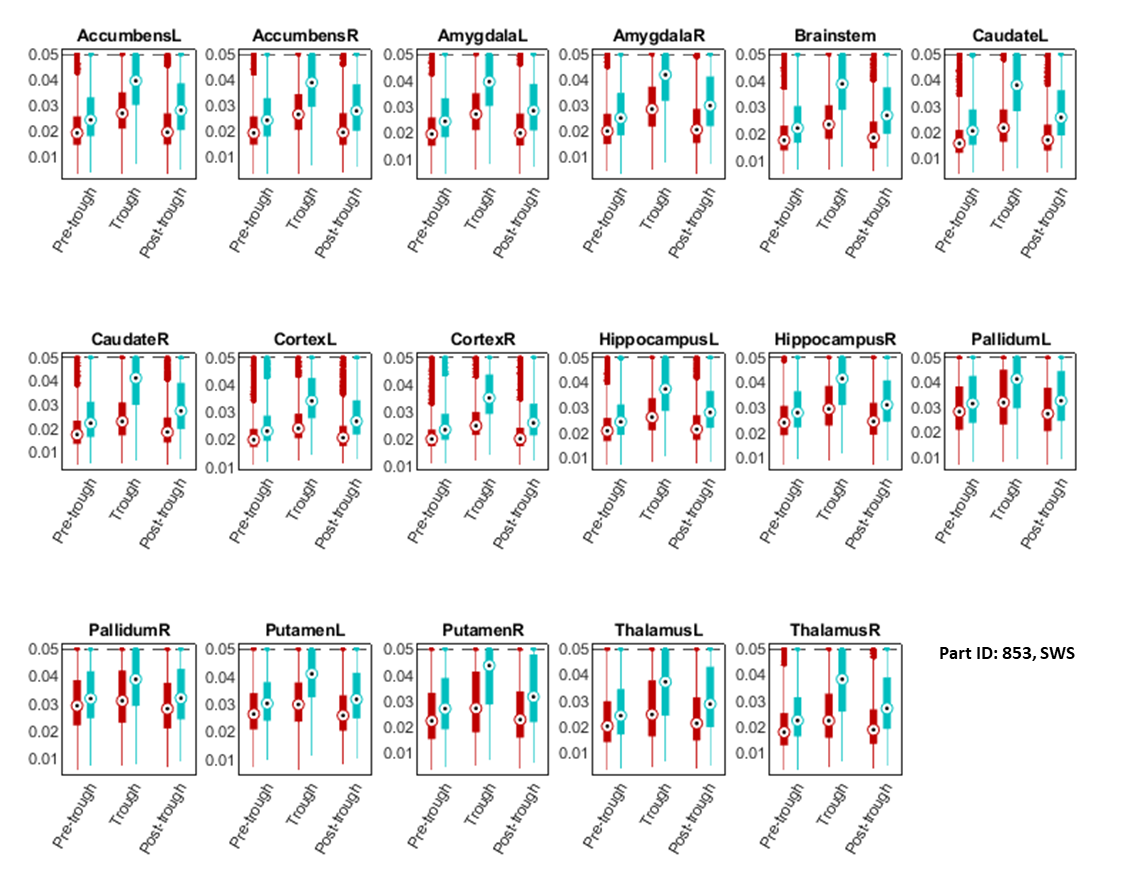


**Fig S2. CSD values for one example participant, separated in Global and non-Global SOs, and by region and time**, **in SWS.** In each panel we show the distribution of CSD values for a given region, separated in time bins before, during and after the trough (labeled “Pre-trough”, “Trough” and “Post-trough”). For every plot, the circle marks the median and thick bars mark the quartiles (25% to 75%). In red, we show the CSD values distribution across all the non-Global SOs detected for this specific participant during their SWS (all SWS SOs found across the sleep night considered). In light blue, we have the analogous plots for Global SOs. Note that for needs of uniform visualization, the values shown are truncated at 0.05. CSD is in arbitrary units. Participant ID 835, SOs found during SWS.

# Figure S3: feature ranking for individual participants, examples.


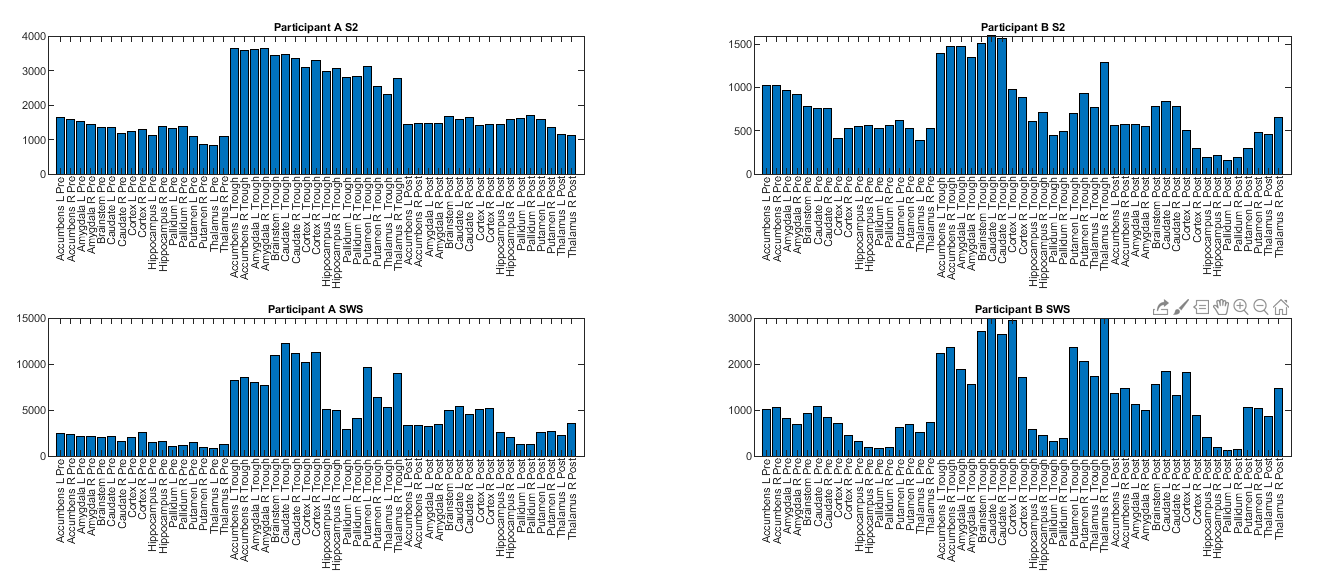


**Fig. S3:** in each panel the x-axis lists all the features of CSD depth profiles, and the y-axis shows the -log(p) feature selection values for that feature in the individual’s dataset. Panels in the left column show feature selection values for one participant (“A”) and the right columns shows values for participant B. Top row shows values for S2 sleep and bottom row shows values for SWS. Note the large difference in y-axis scales, driven by the different count of SOs (Global and non-Global) in each plot.

# Figure S4: feature ranking and selection for S2 SOs


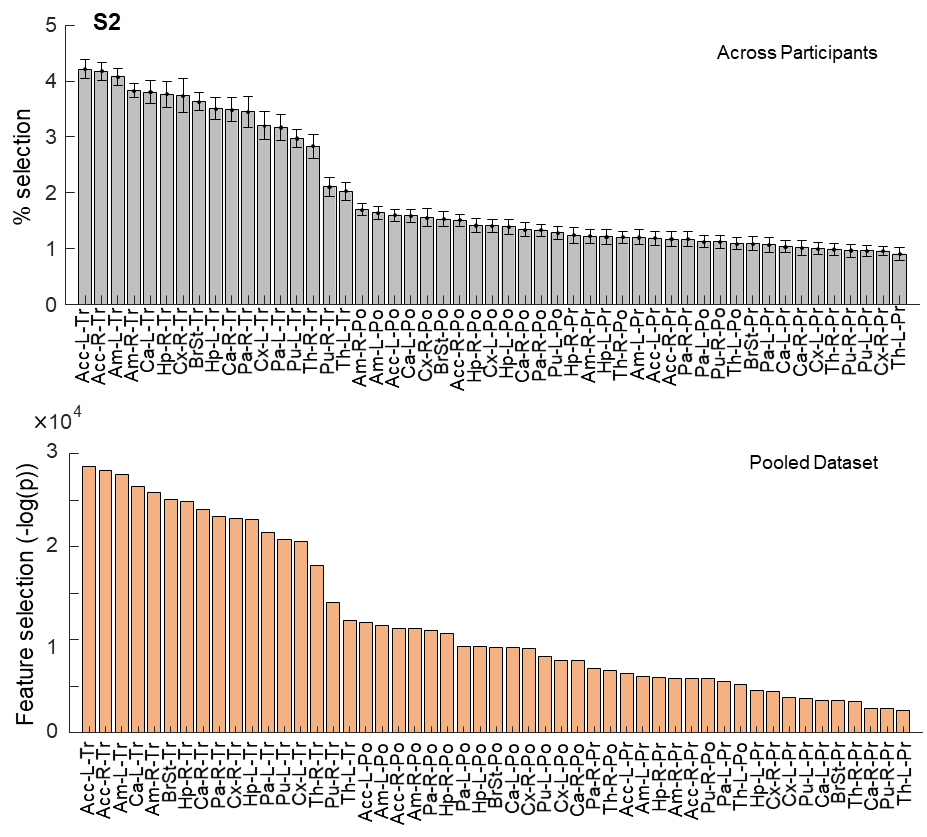


**Fig. S4. Feature ranking for individual and pooled datasets. Considering SO2 detected during S2.** In the top plot, we show the average value among the normalized feature selection values as found for each individual. In the bottom plot, we show the feature ranking resulting from computing feature selection in one single dataset that pooled all the SOs from all individuals. On the x-axis, we label each feature with its region and its time bin. Region labeling is as follows. Acc: nucleus accumbens, Am: amygdala, Ca: caudate nucleus, BrSt: brainstem, Hp: hippocampus, Cx: neocortex, Pa: pallidum, Pu: putamen, Th: thalamus. All regions (except the brainstem) are marked with “R” if in the right hemisphere and “L” for the left. Time bins are identified as Pre-trough (Pr), Post-trough (Po) and at trough (Tr).

# Figure S5: feature ranking and selection for SWS SOs


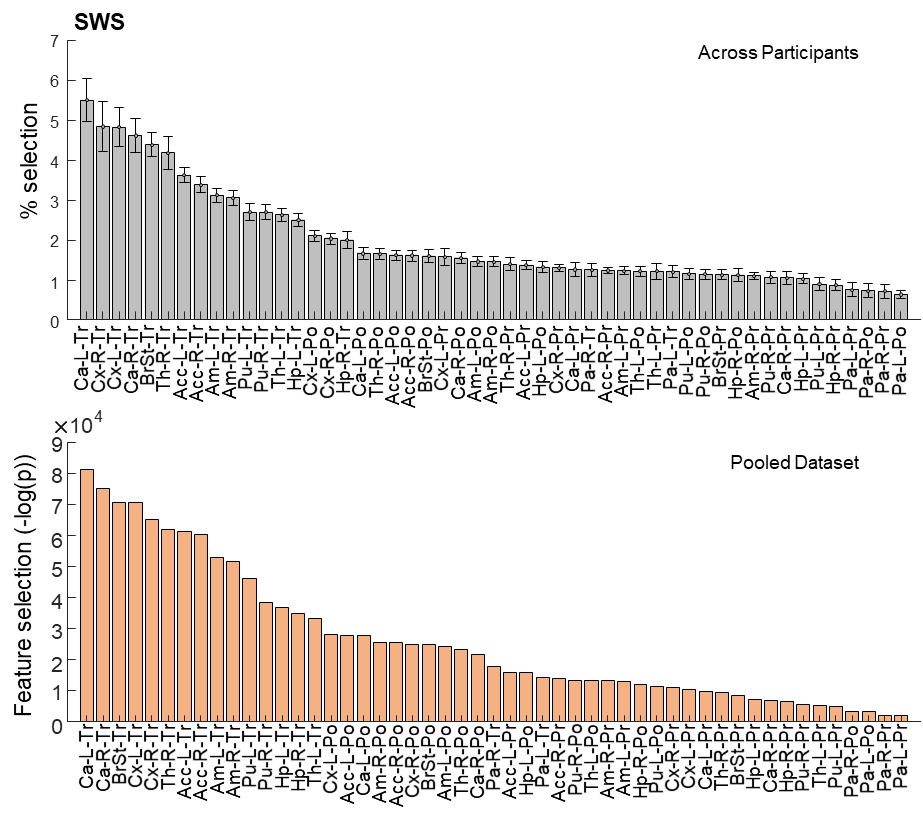


**Fig S5: Feature ranking for individual and pooled datasets. Considering SO2 detected during SWS.** In the top plot, we show the average value among the normalized feature selection values as found for each individual. In the bottom plot, we show the feature ranking resulting from computing feature selection in one single dataset that pooled all the SOs from all individuals. On the x-axis, we label each feature with its region and its time bin. Region labeling is as follows. Acc: nucleus accumbens, Am: amygdala, Ca: caudate nucleus, BrSt: brainstem, Hp: hippocampus, Cx: neocortex, Pa: pallidum, Pu: putamen, Th: thalamus. All regions (except the brainstem) are marked with “R” if in the right hemisphere and “L” for the left. Time bins are identified as Pre-trough (Pr), Post-trough (Po) and at trough (Tr).

# Figure S6: comparing feature ranking derived from individual dataset and the pooled dataset. S2.


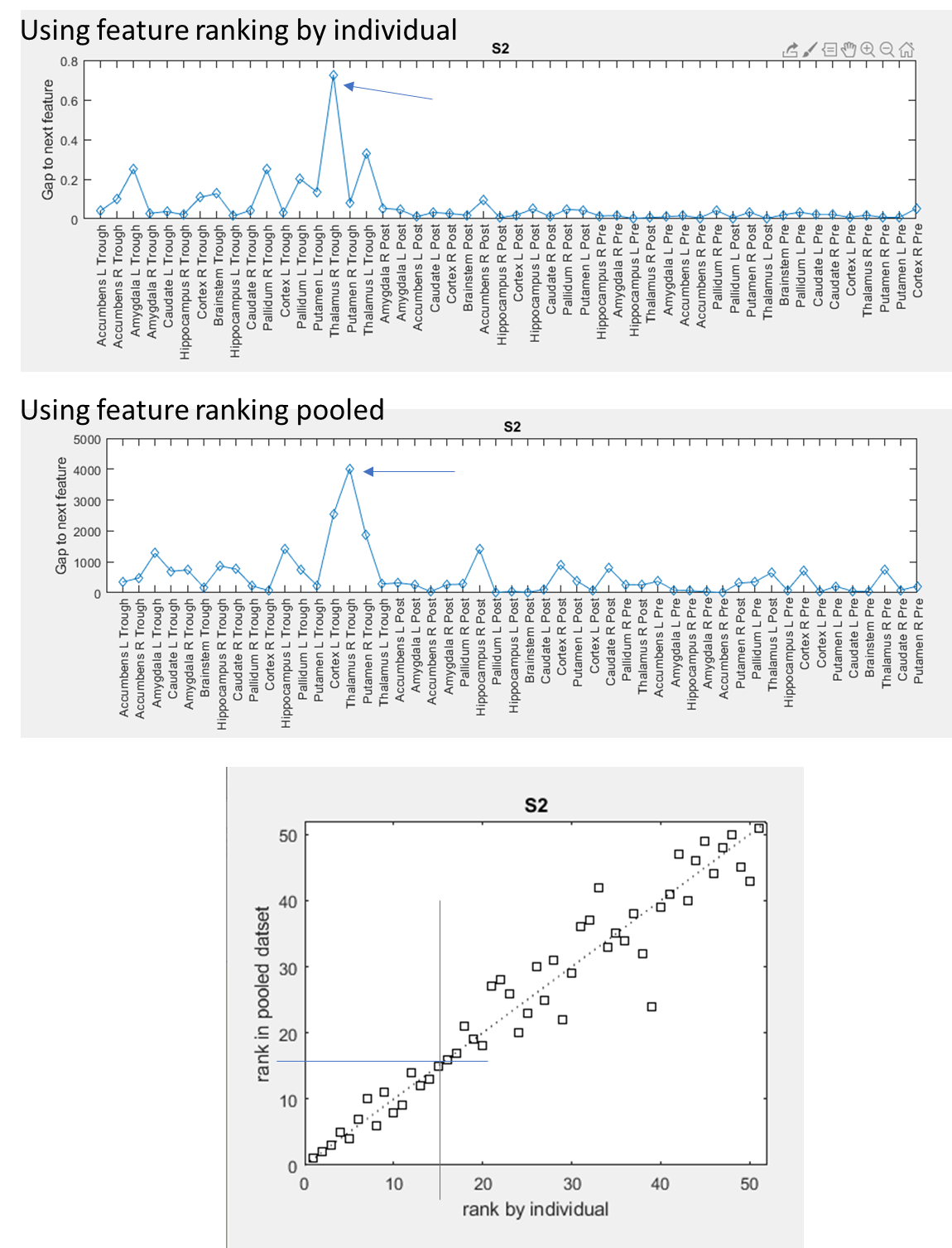
**Fig. S6.** Top plot shows the difference in feature selection values between a given feature and the next feature according to ranking (ranking shown in Fig S4). In other words, this plot shows the size of the steps (a subtraction) between a feature and the next in the histograms of Fig S4 (labeled as “gap to next feature”). This allows an intuitive representation of the degree of differential feature selection values attribute in the histogram of Fig S4. Peaks in the top plot represent large decreases in the feature selection values from the labeled feature to the next one. Features are listed according to the ranking that they obtained in the original histogram. We mark with an arrow the value of high gap, where we choose to separate high-ranking features (according to their selection) from other features. The top plot refers to values in S2 averaged across the normalized feature selection values in individual datasets (i.e., Fig S4 top plot). The middle plot refers to values in S2 found in the pooled dataset (bottom plot of Fig S4). The last plot in this figure compares the rank of each feature in the two domains (across individuals vs pooled dataset) and shows an overall agreement between the two approaches, especially for features ranked above the identified threshold ranks. Blue lines in this plot mark the rank values identified by arrows in the corresponding plots within this figure.

# Figure S7: comparing feature ranking derived from individual dataset and the pooled dataset. SWS.


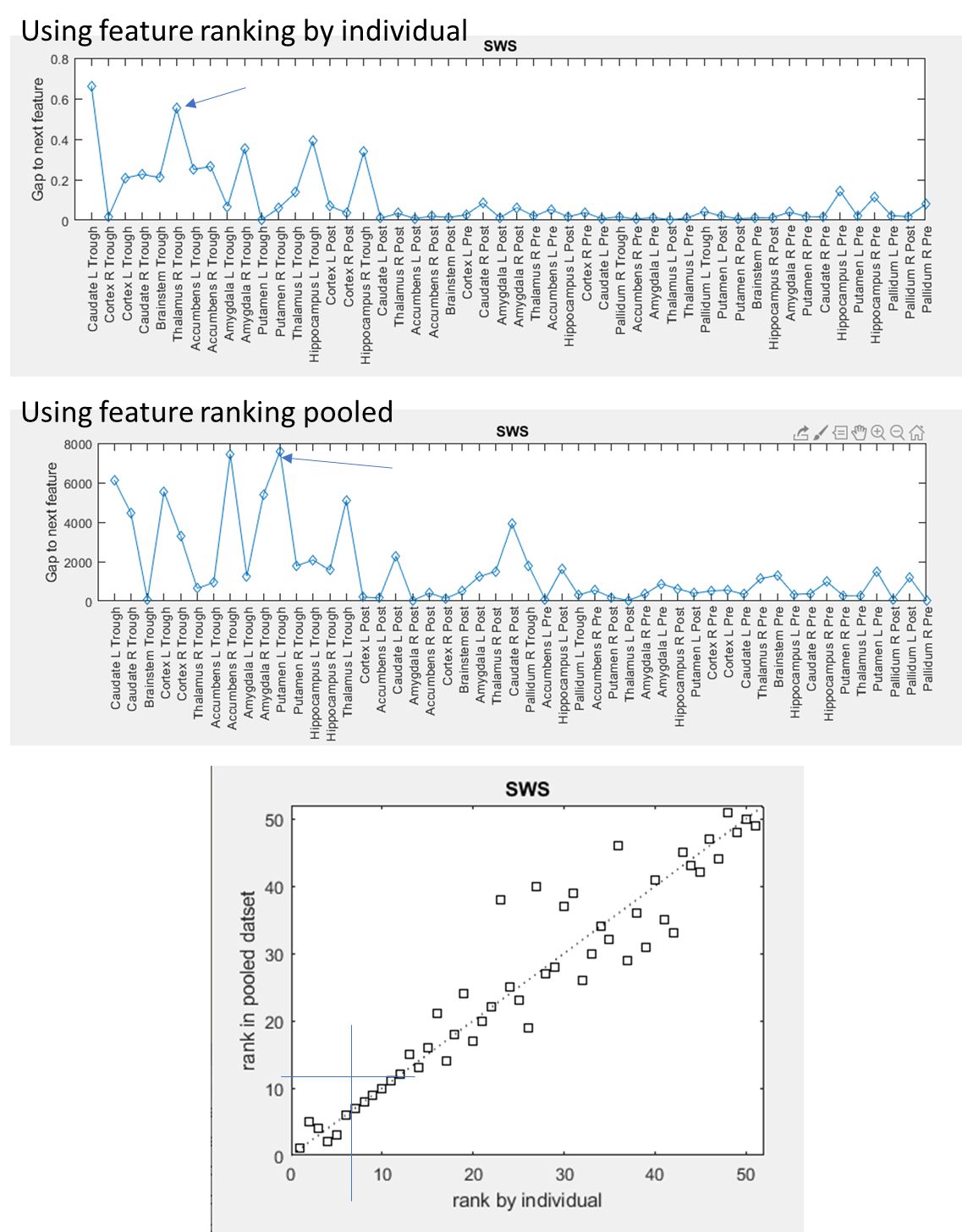
**Fig. S7.** Top plot shows the difference in feature selection values between a given feature and the next feature according to ranking (ranking shown in Fig S5). In other words, this plot shows the size of the steps (a subtraction) between a feature and the next in the histograms of Fig S5 (labeled as “gap to next feature”). This allows an intuitive representation of the degree of differential feature selection values attribute in the histogram of Fig S5. Peaks in the top plot represent large decreases in the feature selection values from the labeled feature to the next one. Features are listed according to the ranking that they obtained in the original histogram. We mark with an arrow the value of high gap, where we choose to separate high-ranking features (according to their selection) from other features. The top plot refers to values in SWS averaged across the normalized feature selection values in individual datasets (i.e., Fig S5 top plot). The middle plot refers to values in SWS found in the pooled dataset (bottom plot of Fig S5). The last plot in this figure compares the rank of each feature in the two domains (across individuals vs pooled dataset) and shows an overall agreement between the two approaches, especially for features ranked above the identified threshold ranks. Blue lines in this plot mark the rank values identified by arrows in the corresponding plots within this figure.

# Performance of ML classification in the pooled dataset: Table

| ML algorithm | S2  51 features | S2  top 15 features | SWS  51 features | SWS  top 11 features | SWS  top 6 features |
| --- | --- | --- | --- | --- | --- |
| FineTree | 0.569 | 0.5294 | 0.496 | 0.5294 | 0.4323 |
| MediumTree | 0.500 | 0.4868 | 0.444 | 0.4868 | 0.4234 |
| CoarseTree | 0.474 | 0.4642 | 0.374 | 0.4642 | 0.3638 |
| LinearDiscriminant | 0.566 | 0.4865 | 0.520 | 0.4865 | 0.3242 |
| QuadraticDiscriminant | 0.552 | 0.3593 | 0.469 | 0.3593 | 0.3873 |
| LogisticRegression | 0.572 | 0.4985 | 0.529 | 0.4985 | 0.3474 |
| GaussianNaiveBayes | 0.409 | 0.4426 | 0.243 | 0.4426 | 0.4011 |
| KernelNaiveBayes | 0.400 | 0.4540 | 0.3453 | 0.4540 | 0.3979 |
| LinearSVM | 0.574 | 0.4885 | 0.519 | 0.4885 | 0 |
| QuadraticSVM | 0.667 | 0.2651 | 0.4155 | 0.2651 | -0.0428 |
| CubicSVM | 0.445 | -0.0257 | -0.0298 | -0.0257 | -0.0166 |
| FineGaussianSVM | 0.985 | 0.8131 | 0.9437 | 0.8131 | 0.5296 |
| MediumGaussianSVM | 0.703 | 0.5901 | 0.6425 | 0.5901 | 0.4574 |
| CoarseGaussianSVM | 0.587 | 0.5316 | 0.5647 | 0.5316 | 0.3993 |
| FineKNN | 1 | 1 | 1 | 1 | 1 |
| MediumKNN | 0.977 | 0.9605 | 0.9698 | 0.9605 | 0.8952 |
| CoarseKNN | 0.704 | 0.6796 | 0.674 | 0.6796 | 0.5797 |
| CosineKNN | 0.976 | 0.9647 | 0.970 | 0.9647 | 0.8842 |
| CubicKNN | 0.978 | 0.9618 | 0.9699 | 0.9618 | 0.8978 |
| WeightedKNN | 1 | 1 | 1 | 1 | 1 |
| BoostedTrees | 0.563 | 0.5173 | 0.513 | 0.5173 | 0.4223 |
| BaggedTrees | 1 | 0.9999 | 1.000 | 0.9999 | 0.9996 |
| SubspaceDiscriminant | 0.531 | 0.4531 | 0.496 | 0.4531 | 0.2610 |
| SubspaceKNN | 1 | 1 | 1 | 1 | 1 |
| RUSBoostedTrees | 0.522 | 0.5084 | 0.461 | 0.5084 | 0.4238 |
| NarrowNeuralNetwork | 0.690 | 0.5910 | 0.619 | 0.5910 | 0.4769 |
| MediumNeuralNetwork | 0.690 | 0.6211 | 0.646 | 0.6211 | 0.4835 |
| WideNeuralNetwork | 0.897 | 0.7057 | 0.698 | 0.7057 | 0.5014 |
| BilayeredNeuralNetwork | 0.695 | 0.6102 | 0.624 | 0.6102 | 0.4832 |
| TrilayeredNeuralNetwork | 0.697 | 0.6200 | 0.622 | 0.6200 | 0.4895 |

**Table S3:** The table shows the MCC values (allowed range (-1,1)) reported in Fig 7 as bars.
